# Morphotropic Phase Boundary in Graft Poly(Vinylidene Fluoride‐*co*‐Tetrafluoroethylene) With High Curie Temperature

**DOI:** 10.1002/advs.74900

**Published:** 2026-03-19

**Authors:** Zekai Fei, Chenyi Li, Yuquan Liu, Yang Li, Yun Zhang, Huamin Zhou, Yang Liu

**Affiliations:** ^1^ State Key Laboratory of Material Processing and Die & Mould Technology, School of Materials Science and Engineering Huazhong University of Science and Technology Wuhan Hubei China; ^2^ Guangdong HUST Industrial Technology Research Institute Guangdong Provincial Key Laboratory of Manufacturing Equipment Digitization Dongguan Guangdong China

**Keywords:** ferroelectric polymers, grafting modification, morphotropic phase boundary, piezoelectricity

## Abstract

Morphotropic phase boundary (MPB) has been demonstrated in various ferroelectric polymers near which piezoelectric coefficient *d*_33_ is substantially enhanced. However, all these ferroelectric polymers contain expensive trifluoroethylene while the Curie temperature *T*_c_ remains low, limiting their practical applications. Here, MPB is explored in trifluoroethylene‐free poly(vinylidene fluoride‐*co*‐tetrafluoroethylene) copolymer by grafting design. We find that grafting induces order‐to‐disorder structural evolution mediated by the formation of MPB reminiscent of composition‐ and irradiation‐induced MPB in trifluoroethylene‐contained ferroelectric polymers. Meanwhile, we show that local conformational disorder is pinned by head‐head defects inherent to tetrafluoroethylene, which is essential to maintain high *T*_c_. Consequently, markedly improved *d*_33_ of −41.6 pC N^−1^ and high *T*_c_ of over 130°C are concurrently achieved in graft poly(vinylidene fluoride‐*co*‐tetrafluoroethylene) copolymers, outperforming currently existing ferroelectric polymers with high *T*_c_. These findings add a trifluoroethylene‐free member into MPB family, which offers a solution to address the long‐standing inverse *d*_33_‐*T*_c_ relationship in ferroelectric materials.

## Graphical abstract

The first TrFE‐free ferroelectric polymers exhibiting concurrently a morphotropic phase boundary and high Curie temperature are reported through a grafting strategy, which is completely different from previous methods to design MPB by composition and irradiation. This finding offers a cost‐effective solution to decode the long‐standing inverse relation between piezoelectricity and Curie temperature in ferroelectric materials.

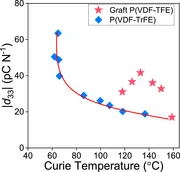

## 1 Introduction

Flexible and light‐weight ferroelectric polymers are promising choices of materials to develop flexible and wearable sensors, transducers, and actuators with desired shapes [1, 2]. Pioneering studies [3, 4, 5, 6, 7, 8, 9, 10, 11] concentrated on the pursuit of the high‐polar *β* phase (all‐*trans* conformation) with high Curie temperature *T*_c_. However, owing to the inverse relation between piezoelectric coefficient *d*_33_ and *T*_c_, these ferroelectric polymers often exhibit unsatisfied *d*_33_. For instance, ferroelectric poly(vinylidene fluoride‐*co*‐trifluoroethylene) (P(VDF‐TrFE)) [3, 7] and poly(vinylidene fluoride‐*co*‐tetrafluoroethylene) (P(VDF‐TFE)) [7, 12] copolymers with high VDF content (>80 mol%) exhibit a small *d*_33_ of about ‐20 pC N^−1^ while preserving a high *T*_c_ above 110°C. Different from P(VDF‐TrFE) and P(VDF‐TFE) copolymers, PVDF homopolymer adopts the non‐polar the *α* phase (TGTḠ conformation, T, *trans*, G, *gauche*) as the ground state, which necessarily requires mechanical stretching to induce the transition into the polar *β* phase [13, 14]. Stretched PVDF homopolymers with a typical *d*_33_ of about −30 pC N^−1^ and a high *T*_c_ above 170°C may undergo stretched (polar) to unstretched (non‐polar) transformation upon heating, despite high *T*_c_, resulting in degradation of *d*_33_ at elevated temperatures. Consequently, it remains challenging to overcome the longstanding inverse *d*_33_‐*T*_c_ relation in ferroelectric polymers.

Different from previous studies on high fraction of the polar *β* phase [15, 16], the morphotropic phase boundary (MPB) describing the transition region with coexisting phases with similar energies has been used to enhance piezoelectric response [14]. The generation of energetically nearly degenerate crystalline phases is often achieved at the expense of lowered *T*_c_ typically below 90°C [17, 18, 19, 20, 21, 22, 23, 24]. Meanwhile, till now there are only a few ferroelectric polymers exhibiting MPB behavior [17, 18, 19, 20, 21, 22, 23, 24] while all these polymers contain chiral TrFE, which is critically related to the presence of strong local conformational disorder [25]. Massive use of TrFE is limited by high cost and safety concern. This makes synthesis of TrFE‐based ferroelectric polymers inaccessible to most academic labs [14]. Consequently, alternative frameworks beyond conventional methods are urgently needed to construct TrFE‐free MPB combined with a high *T*_c_, which is desired for boosting practical piezoelectric applications.

To address this challenge, here MPB is designed in graft P(VDF‐TFE) copolymers without the need for TrFE. Differing from chiral TrFE, inclusion of TFE introduces ‐CF_2_‐CF_2_‐ units corresponding to head‐head (HH) defects, which were believed to be crucial to smear the energy barriers between different chain conformations, leading to stabilization of the ordered ferroelectric phase against the disordered paraelectric phase in P(VDF‐TFE) copolymers [26, 27, 28, 29, 30, 31, 32, 33, 34, 35]. In this regard, grafting reactions induce the reduction in VDF content through the dehydrofluorination [36] yielding increased relative content of HH defects. This may help to tune the relative stability between different chain conformations. It may offer a new degree of freedom to enable order‐to‐disorder phase evolution mediated by the formation of MPB reminiscent of that achieved by the compositional approach in TrFE‐based ferroelectric polymers [17, 18, 19, 20, 21, 22, 23, 24]. As a result, a substantially improved *d*_33_ may be achieved near MPB while preserving high *T*_c_ due to pinning of local conformational disorder by HH defects in graft P(VDF‐TFE) copolymers. Our work may therefore unlock the door to design of novel MPB with high *T*_c_ in TrFE‐free ferroelectric polymers, which offers a cost‐effective solution to decode the long‐standing *d*_33_‐*T*_c_ relation in ferroelectric materials [37].

## 2 Results and Discussion

**FIGURE 1:**
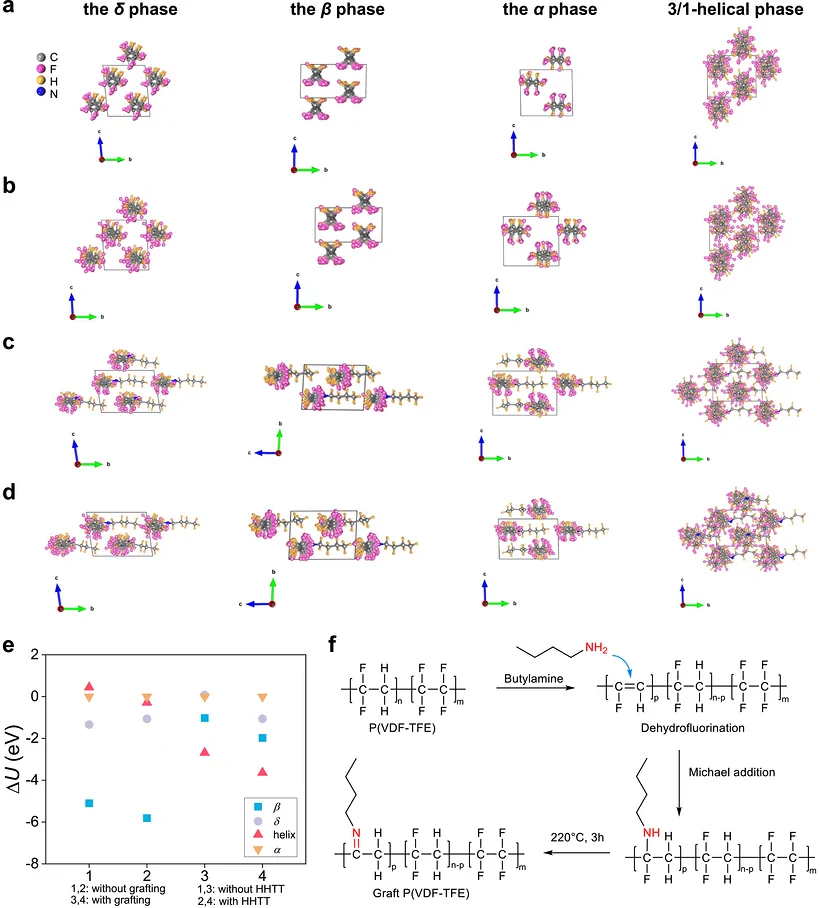
(a) Pristine P(VDF‐TFE) of different chain conformations without HHTT defects. (b) Pristine P(VDF‐TFE) of different chain conformations with HHTT defects. (c) Graft P(VDF‐TFE) of different chain conformations without HHTT defects. (d) Graft P(VDF‐TFE) of different chain conformations with HHTT defects. In (a)–(d), the *δ*, *β*, *α*, and 3/1‐helical phases are listed from the left to the right. (e) The relative energy diagram considering different crystalline phases with inclusion of HH defects, HHTT defects and grafting. The numbers (1–4) correspond to structures: (1) Pristine P(VDF‐TFE) without HHTT defects a). (2) Pristine P(VDF‐TFE) with HHTT defects (b). (3) Graft P(VDF‐TFE) without HHTT defects (c). (4) P(VDF‐TFE) with HHTT defects (d). (f) Scheme of grafting reactions in P(VDF‐TFE) with butylamine.

We focus on the high‐VDF region of the phase diagram of P(VDF‐TFE) different from equimolar region in P(VDF‐TrFE). Previous potential‐energy calculations predicted that the ferroelectric *β* phase may be energetically more favorable than the paraelectric *α* phase as the HH defects are above 8 mol% with the concurrent presence of head‐head/tail‐tail (HHTT) defects at a concentration of 5 mol% [24]. Provided PVDF exhibits rich crystalline phases [14], only the *β* and *α* phases were considered [26]. To gain further structural insight into relative energetic stability in P(VDF‐TFE), we performed density‐functional theory (DFT) calculations, whereas four typical crystalline phases were considered, including the *β*, *α, δ*, and 3/1‐helical ((TG)_3_ or (TḠ)_3_) phases (Figure 1a–d, Figures S1–S4 and Table S1). In our calculations, we choose an HHTT defect content of 5 mol% which is typical according to previous works [26, 38]. In this regard, the P(VDF‐TFE) composition corresponds to 95/5 mol%. In our calculations, we use the energy of the *α* phase as the reference, the relative energy of other phases with respect to this phase is used to define the relative energy stability. Such method has been frequently used to study the phase evolution in ferroelectric polymers [17, 25]. With the absence of HHTT defects, the energy of different crystalline phases from the lowest to the highest follows the sequence: Δ*U_β_<*Δ*U_δ_<*Δ*U_α_<*Δ*U*_helix_ (Figure 1e). With the presence of HHTT defects, such energy relation is switched to: Δ*U_β_<*Δ*U_δ_<*Δ*U*_helix_ *<*Δ*U_α_* whereas the energy of 3/1‐helical phase is lowered compared with the *α* phase (Figure 1e). These theoretical results are consistent with previous results [26] showing that the *β* phase is the ground state due to incorporating TFE. Moreover, our results reveal the presence of very small energetic barriers between the *α* phase and other crystalline phases, which has been out of reach from previous results [26]. For instance, we find that the energetic difference between the 3/1‐helical phase and the *α* phase is as small as 0.3 eV for P(VDF‐TFE) with HHTT defects (Figure 1e). This result suggests that the paraelectric‐like phase presented in P(VDF‐TFE) may adopt other phases instead of the *α* phase when incorporating chemical modification such as grafting, which may even compete with the *β* phase, leading to the formation of MPB.

To confirm this physical picture, we focus on graft P(VDF‐TFE) by butylamine. Interestingly, our calculation results show that in graft P(VDF‐TFE), the 3/1‐helical phase exhibits lower energy than the *β* phase and the *α* phase, yielding the energy sequence as Δ*U*_helix_ *<*Δ*U_β_<*Δ*U_α_<*Δ*U_δ_* without HHTT defects and Δ*U*_helix_ *<*Δ*U_β_<*Δ*U_δ_<*Δ*U_α_* with HHTT defects (Figure 1e). This result therefore demonstrates that after grafting, the ground state of P(VDF‐TFE) is switched to the 3/1‐helical phase rather than the *β* phase in the pristine counterpart, which occurs independent of HHTT defects. This finding explicitly elucidates that grafting as a leverage to tune the energetic competition between different chain conformations leading to the stabilization of the 3/1‐helical phase rather than the *α* phase. Such phase evolution induced by grafting in P(VDF‐TFE) is like that tuned by the spatial arrangement of chiral defects which offers an alternative route to construct MPB [17, 25]. Note that the specific P(VDF‐TFE) predicted by our DFT calculations may be slightly varied when non‐idealized defects are considered. However, the predicted phase evolution by grafting should be maintained.

**FIGURE 2:**
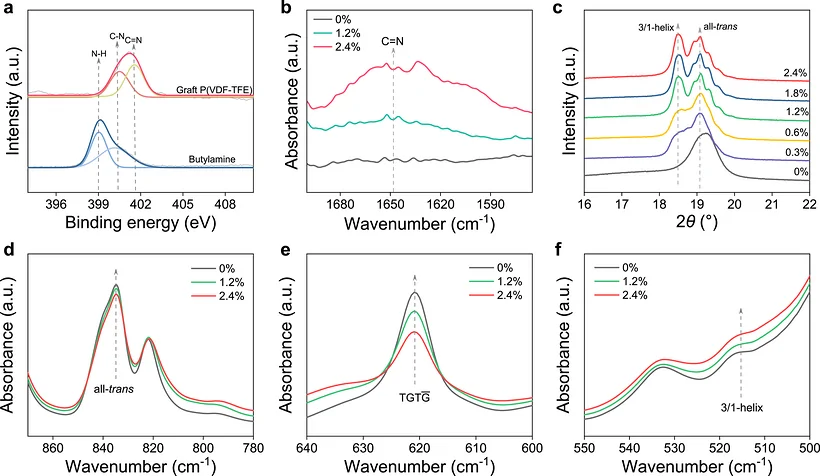
(a) N1s XPS result. (b) FTIR spectra whereas the dashed line indicates the characteristic band corresponding to C═N bond. (c) XRD result. (d) Characteristic infrared band at around 835 cm^−1^ corresponding to all‐*trans* conformation. (e) Characteristic infrared band at around 620 cm^−1^ corresponding to TGTḠ conformation. (f) Characteristic infrared band at around 515 cm^−1^ corresponding to 3/1‐helix conformation.

To confirm the theoretical prediction on grafting‐induced MPB in P(VDF‐TFE), graft P(VDF‐TFE) 95/5 mol% copolymers with various grafting densities were synthesized by using butylamine as the grafting agent, following the scheme for grafting reactions (Figure 1f). C═C double bonds are induced through dehydrofluorination followed by Michael addition reaction [36], which enables grafting of amine onto P(VDF‐TFE) through C═N double bonds. The formation of graft structure is supported by the existence of C═N double bonds according to X‐ray photoelectron spectroscopy (XPS) and Fourier‐transform infrared (FTIR) spectroscopy results. For instance, graft P(VDF‐TFE) shows the appearance of a characteristic peak near 402 eV, which is absent in the pristine counterpart in XPS data (Figure 2a). Meanwhile, the presence of C═N double bonds is also supported by the characteristic infrared band near 1648 cm^−1^ (Figure 2b). Our swelling experimental results indicate the absence of crosslinking structure for the grafting density below 2.4% (Figure S5).

Our structural characterization by X‐ray diffraction (XRD) in Figure 2c shows that pristine solution‐processed films exhibit a singlet at 2*θ*═19.2° indicative of the *β* phase. The singlet is asymmetric with the intensity at lower 2*θ* larger than that at higher 2*θ*. This may suggest the presence of mixed phases instead of a single polar phase, which agrees with our DFT results and previous results [26]. Interestingly, we find that the singlet in pristine P(VDF‐TFE) is split into a doublet in graft counterparts (Figure 2c). The newly formed peak is located near 18.5° indicative of the presence of a disordered 3/1‐helical phase according to our DFT calculations, which develops with increasing the grafting density at the expense of peak density locating at higher 2*θ*═19.1°. Such grafting‐induced phase evolution is like that occurring in previous works on composition‐ and irradiation‐induced MPB [17, 18, 19, 20, 21, 22, 23, 24]. This strongly indicates the existence of an order‐to‐disorder phase transition, while near the critical grafting density with nearly the same diffraction densities, both ordered and disordered crystalline phases are nearly energetically degenerate [17, 18, 19, 20, 21, 22, 23, 24]. Consequently, our combined theoretical and structural results elucidate that grafting may enable the formation of MPB without the need for chiral defects, which is beyond the previous compositional framework [17, 25].

FTIR spectra (Figure S6) not only collaborate with XRD results but also strongly support that the disordered phase may adopt a disordered helical chain conformation like that occurring in compositionally induced MPB in P(VDF‐TrFE) copolymers [17, 25] and P(VDF‐TrFE‐CTFE) terpolymers [18, 39]. For instance, upon grafting, a decrease in the infrared band at around 835 cm^−1^ characteristic of all‐*trans* conformation is observed (Figure 2d). This is accompanied by a marked decrease in the infrared band at around 620 cm^−1^ assigned to TGTḠ conformation (Figure 2e). This result may help to rule out the *α* phase as the origin of the disordered phase. Moreover, it is found that as the grafting density increases, the characteristic band at around 515 cm^−1^ corresponding to 3/1‐helix conformation develops in its intensity, indicative of stabilization of 3/1‐helix caused by grafting (Figure 2f). Meanwhile, the broadening of the infrared band at around 515 cm^−1^ is found (Figure 2f), reminiscent of P(VDF‐TrFE) with decreasing VDF content [17], which further implies the formation of disordered phase. Consequently, these structural results indicate the existence of *trans*/helix phase boundary leveraged by grafting strategy, which resembles that occurring in composition‐ and irradiation‐induced MPB [17, 18, 19, 20, 21, 22, 23, 24].

**FIGURE 3:**
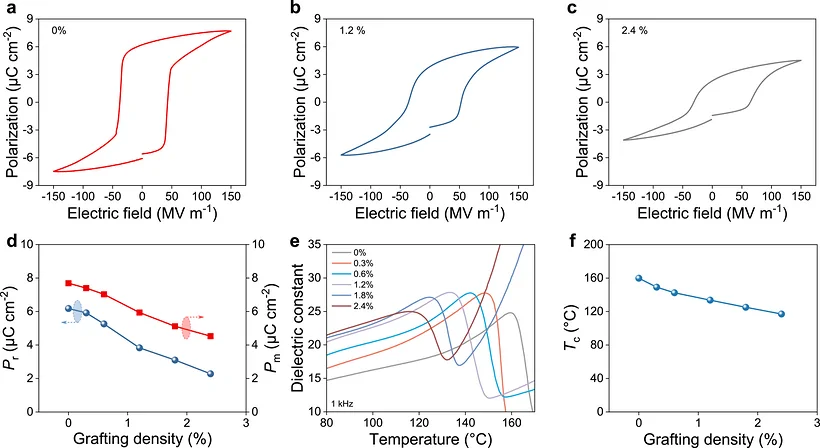
*P–E* loop results in graft P(VDF‐TFE) with various grafting densities (10 Hz). (a) 0%. (b) 1.2%. (c) 2.4%. (d) *P*_r_ and *P*_m_ as a function of grafting density. (e) Temperature‐dependent dielectric constant at 1 kHz under different grafting densities. (f) *T*_c_ deduced from dielectric result as a function of grafting density.

The destabilization of long‐range ferroelectric instability is evaluated by polarization‐electric field (*P–E*) hysteresis loops (Figure 3a–c, Figures S7–S9). Pristine P(VDF‐TFE) is characterized by a typical ferroelectric type *P–E* loops with a large remnant polarization *P*_r_ of 6.2 µC cm^−2^ indicative of the existence of long‐range ferroelectric distortion, which is consistent with the structural result (Figure 2c). *P–E* loops in graft P(VDF‐TFE) evolves into pinched or antiferroelectric‐like type as the grafting density increases. Such type of *P–E* loops in ferroelectric polymers, including history and mechanism has been reviewed in [1], which is not relevant to the formation of the antiferroelectric phase. Instead, the pinched type often occurs when disordered relaxor or paraelectric‐like phase appears and grows [17, 21, 25]. With increasing the grafting density, it is found that *P*_r_ and the maximum polarization *P*_m_ decrease dramatically (Figure 3d) stemming from order‐to‐disorder phase transition induced by grafting, which also agrees with XRD results (Figure 2c). Note that the observed *P–E* loops are not perfectly closed, which often occur in ferroelectric polymers, especially at high fields well above the coercive field [1, 14]. The non‐close shape can be attributed to the presence of charged defects and charge injections from the electrode‐polymer interfaces (Figure S10). We show the presence of high stability of *d*_33_ with electrical and mechanical stimuli (Figures S11 and S12), indicating their negligible contributions to piezoelectric applications.

The temperature‐dependent phase transition behavior is studied by differential scanning calorimetry (DSC) and dielectric spectra. We find the absence of the endothermic peak in DSC heating scans until the melting point *T*_M_ (Figure S13), while the dielectric peak at high temperatures is attributed to the melting behavior instead of ferroelectric‐to‐paraelectric phase transition (Figures S14–S19). Therefore, despite the disorder caused by grafting, graft P(VDF‐TFE) 95/5 mol% maintains a high *T*_c_ above *T*_M_. This is one key reason why the strong dielectric relaxation‐characteristic of relaxor behavior is not observed in the temperature‐dependent dielectric spectra (Figures S14–S19). *T*_c_, where the dielectric anomaly appears in the temperature‐dependent dielectric diagram, shifts towards lower temperature as the grafting density increases (Figure 3e,f). Such a shift in *T*_c_ towards lower temperatures may result from the grafting‐induced destabilization of long‐range ferroelectric instability (Figure 2c). The presence of conformational disorder caused by grafting is further supported by a broader dielectric peak driven by grafting (Figure 3e), indicating that the phase transition near *T*_c_ is diffused due to grafting.

**FIGURE 4:**
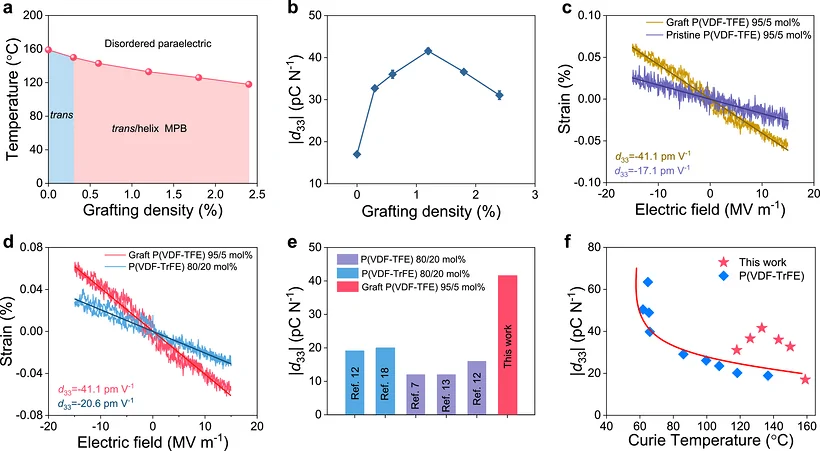
(a) Temperature‐grafting density phase diagram. MPB refers to *trans*/helix coexisting region (0.3%–2.4%) separating *trans*‐planar and 3/1‐helical phases whereas *trans*‐planar phase is normal ferroelectric and 3/1‐helical phase is paraelectric‐like. (b) *d*_33_ as a function of grafting density. (c) Electric field‐induced strain between graft and pristine P(VDF‐TFE). The slope corresponds to the converse piezoelectric coefficient *d*_33_. The grafting density is 1.2%. (d) Electric field‐induced strain between graft P(VDF‐TFE) and P(VDF‐TrFE) 80/20 mol%. (e) Comparison of *d*_33_ between different ferroelectric polymers with high VDF content. (f) *d*_33_ versus Curie temperature. The star refers to graft P(VDF‐TFE) with a grafting density of 1.2%. The red line indicates the inverse relation between *d*_33_ and *T*_c_.

Figure 4a presents the temperature‐grafting density phase diagram, whereas MPB region corresponds to a grafting density range from 0.3% to 2.4%. The right side of MPB region is assigned to 2.4% not due to the formation of crosslinking above 2.4% (i.e., 3% in Figure S20), which may overlap with grafting regime in the phase diagram [36]. Instead, such assignment is based on *P–E* loop and XRD results. For instance, the existence of pinched *P–E* loops observed for 2.4% at 100 MV m^−1^ (Figure 3c) is regarded as the signature of the stabilization of relaxor behavior [1, 14, 17, 18, 20]. Meanwhile, the relative peak density assigned to 3/1‐helical phase exceeds that corresponding to *trans*‐planar phase for 2.4% (Figure 2c), suggesting the destabilization of long‐range ferroelectric instability above 2.4%. This is crucial to the MPB concept as it represents the first example of TrFE‐free MPB in ferroelectric polymers [14], which is driven by grafting instead of composition or irradiation.

The existence of MPB in graft P(VDF‐TFE) is confirmed by piezoelectric measurements. *d*_33_ of P(VDF‐TFE) with various VDF content has been reported with the |*d*_33_| values typically smaller than 20 pC N^−1^ ([7, 12, 40, 41] and Table S2). Pristine P(VDF‐TFE) 95/5 mol% exhibits a *d*_33_ of ‐17.0 pC N^−1^ measured by *d*_33_ meter through the direct piezoelectric effect (Figure 4b, Figures S21–S23), which is well consistent with the *d*_33_ of −17.1 pm V^−1^ measured by electric‐field induced strain through the converse piezoelectric effect (Figure 4c). |*d*_33_| of pristine P(VDF‐TFE) 95/5 mol% is slightly smaller than that (about 20 pC N^−1^) of benchmark P(VDF‐TrFE) 80/20 mol% with a lower *T*_c_ (Figure 4d, Figure S24). Moreover, we find that |*d*_33_| of graft P(VDF‐TFE) measured by *d*_33_ meter is markedly enhanced reaching the maximum value of 41.6 pC N^−1^ at a grafting density of 1.2% (Figure 4b, Figures S25–S27). The existence of large *d*_33_ is verified by electric‐field induced strain, whereas the slope of linear strain response yields a *d*_33_ of −41.1 pm V^−1^ (Figure 4c). This corresponds to over 140% enhancement over pristine counterpart which also exceeds previous results obtained in P(VDF‐TFE) copolymers [7, 12, 40] (Figure 4e, Table S2). Note that our results are entirely distinct from previous grafting studies, whereas improved piezoelectric properties of ferroelectric polymers and nanocomposites is mainly achieved based on the grafting‐induced stabilization of all‐*trans* conformation [42, 43] (For comparison, *d*_33_ of graft PVDF is only ‐17 pC N^−1^). Consequently, piezoelectric results elucidate the formation of MPB caused by the grafting strategy near which *d*_33_ is markedly improved.

Our methods to demonstrate the MPB in graft P(VDF‐TFE) are in line with well‐established practices in the field of MPB in ferroelectric polymers [17, 18, 19, 20, 21, 22, 23, 24]. The formation of MPB does not contradict continuous conformational disordering or relaxor‐like ferroelectric behavior as the appearance of MPB region is critically linked with the emergence of conformational disorder and such phase evolution is typically not sharp but continuous [17, 18, 19, 20, 21, 22, 23, 24]. Our DFT results rationalize the underlying link between grafting and the emergence of phase competition which is confirmed by the experimental results. The presence of grafting‐induced conformational disorder is also demonstrated by our observations. Based on our findings in this work, further structural information regarding grafting sites, defect distributions, and their influence on crystalline phase stability is desirable, which can be addressed in future studies. In addition, other factors such as mechanical softening, changes in elastic modulus, local dipolar contributions introduced by grafting, or nonlinear electromechanical effects can also influence *d*_33_ response. However, most of these factors have been included in MPB framework. For instance, the mechanical softening and change in elastic modulus have been considered as a result of MPB behavior, which yields a good agreement with experimental results [17, 22]. As we mentioned above, the local dipolar contributions introduced by grafting, which is also included in MPB framework [17, 25] are also key to the design of MPB and thus enhanced *d*_33_ response. Nonlinear electromechanical effects may play a minor role here as we mainly focus on linear piezoelectric regime in our work.

Figure 4f summarizes |*d*_33_|‐*T*_c_ relationship in P(VDF‐TrFE) with different VDF content, whereas the results obtained in this work are also provided. An inverse dependence of |*d*_33_| on *T*_c_ is illustrated, whereas substantially enhanced *d*_33_ is achieved by compositionally induced MPB approach at the expense of considerably lowered *T*_c_ in ferroelectric polymers (Table S2) [17, 18, 19, 20, 21, 22, 23, 24]. We note that the inverse relationship between *d*_33_ and *T*_c_ not only occurs in ferroelectric polymers such as P(VDF‐TrFE) but also holds for inorganic ferroelectric materials [38]. Indeed, the inverse relation is usually considered as the empirical behavior without a universal formal equation. In this regard, we find that *d*_33_ of graft P(VDF‐TFE) notably deviates from the inverse relation (Figure 4f). Such deviation occurring in the graft copolymer is not present in pristine ferroelectric polymers, which suggests that grafting is mainly responsible for the deviation from the inverse relation. As grafting makes the energetic barriers between competing chain conformations smaller, it enables greatly enhanced *d*_33_ while preserving high *T*_c_ due to the presence of HH defects. As a result, the largest deviation occurs in the grafting density of 1.2% (Figure 4f). This finding not only indicates that MPB revealed in graft P(VDF‐TFE) at the high VDF region is completely distinct from that obtained in P(VDF‐TrFE) near the equimolar ratio between VDF and TrFE [17] but also offers a compelling solution to break the inverse relation commonly occurring in ferroelectrics [37]. The price of TFE is only about 4% of TrFE which corresponds to about 20% of VDF. As the synthesis of P(VDF‐TFE) is much more cost‐effective and safer compared to P(VDF‐TrFE), our results may inspire large‐scale fabrication of graft P(VDF‐TFE) copolymers for various piezoelectric applications. For industrial fabrication, other process methods without the use of harmful solvents can be considered such as extrusion process while solution process may require costly solvent recovery system. Fluorine‐free polymers could also be explored in future works which can expand the scope of grafting technique in improving their piezoelectric response. In addition, the temperature‐dependent *d*_33_ results further confirm the high *T*_c_ while high stability of *d*_33_ is observed upon electrical cycles (Figure S12).

**FIGURE 5:**
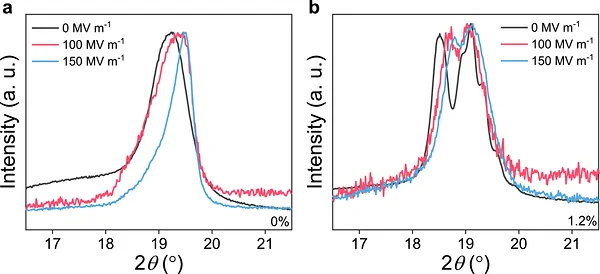
(a) Electric field‐dependent XRD in pristine P(VDF‐TFE). (b) Electric field‐dependent XRD in graft P(VDF‐TFE) with a grafting density of 1.2%.

To gain deeper insight into enhanced *d*_33_ in graft P(VDF‐TFE) copolymers, electric‐field dependent XRD is performed. We find that the asymmetric peak pattern shown in pristine P(VDF‐TFE) without electric field evolves into symmetric‐like pattern under electric fields, accompanied by a notable shift towards higher 2*θ* (Figure 5a). The peak becomes pronouncedly sharp under 150 MV m^−1,^ supporting the depression of the weak minor phase in pristine P(VDF‐TFE). This is also in line with our DFT results and previous results [26] where the electric field makes the long‐range ferroelectric phase energetically more favorable. For graft P(VDF‐TFE), we show that the peak locating at lower 2*θ* characteristic of disordered helical phase shifts towards higher 2*θ* values which is accompanied by the decrease in the peak intensity as the electric field increases (Figure 5b). In this regard, the peak evolution from the doublet into a singlet is not observed in graft P(VDF‐TFE) even at a high electric field of 150 MV m^−1^ which is different from a recent result on irradiation‐induced MPB in P(VDF‐TrFE) [21]. Despite these differences, the polarization rotation mechanism caused by electric field‐induced phase transition is responsible for improved *d*_33_ similar with that in composition‐driven MPB in P(VDF‐TrFE) copolymers [44].

The delayed electric‐field induced phase transition in graft P(VDF‐TFE) may be critically dependent on the unique feature of TFE unit as the local conformational disorder may be pinned by the presence of local HH defects which requires higher energy input to trigger local bond rotation due to steric hindrance stemming from TFE units. Evidence to support delayed phase transition caused by HH defect can be found in *P–E* loop results, which show considerable increase in coercive field *E*_c_ with increasing the grafting density (Figure 3a–c, Figures S7–S9 and S28). Therefore, both *P–E* loop and electric field‐dependent XRD results suggest that the local conformational disorder is pinned by HH defects. This unique feature makes HH defects completely distinct from the counterparts occurring in P(VDF‐TrFE) mainly dominated by chiral defects [17] and irradiated P(VDF‐TrFE) tuned by irradiation‐induced chemical defects [21]. Both of the latter two modified P(VDF‐TrFE) exhibit a notable decrease in *P*_r_ and *E*_c_ with increasing the local conformational disorder [17]. These contrasting results suggest HH defects may be essential to enable stable *d*_33_ response with respect to temperature. Future theoretical studies are highly desired to provide further fundamental insights into the correlation between local conformational disorder and *d*_33_ response in graft P(VDF‐TFE), which may offer promising opportunities for designing high‐*T*_c_ ferroelectric polymers with large *d*_33_.

## 3 Conclusion

In summary, based on our previous findings, our current work reveals that grafting may drive the phase transition from all‐*trans* to 3/1‐helix conformation mediated by the formation of previously unidentified MPB. This is fully consistent with the main definition of MPB revealed by changing the polymer composition. We demonstrate the grafting‐induced MPB behavior in P(VDF‐TFE) without TrFE as required in previous MPB in ferroelectric polymers. This leads to a markedly enhanced *d*_33_ of −41.6 pC N^−1^ exceeding that of the benchmark P(VDF‐TrFE) with high *T*_c_. The unique role of HH defect inherent to TFE in pinning the induced local conformational disorder is revealed, which is essential to maintain a high *T*_c_ of over 130°C. The design of MPB via an alternative grafting method and the realization of MPB in TrFE‐free polymer composition both highlight the potential by integrating MPB concept and polymer physics/chemistry in inspiring further optimization of piezoelectric properties for practical applications.

## 4 Experimental Section

### 4.1 Preparation of Grafted P(VDF‐TFE) Films

P(VDF‐TFE) with the VDF/TFE molar ratio of 95/5 mol% and P(VDF‐TrFE) with the VDF/TrFE 80/20 mol% were purchased from PolyK Technologies and Piezotech at Arkema, respectively. Butylamine and *N*,*N*‐Dimethylformamide were purchased from Sigma‐Aldrich. All chemicals were utilized without modifications. Pristine P(VDF‐TFE) was dissolved in *N*,*N*‐Dimethylformamide at a concentration of 60 mg mL^−1^ under continuous agitation at room temperature for 12 h. Butylamine with various masses was then introduced into the polymer solution, followed by homogenization at 45°C under continuous stirring for 3 h. The resulting mixture was cast onto glass substrates and dried at 60°C for 8 h to evaporate the solvent and subsequently annealed at 220°C for 3 h to enable grafting reactions. After cooling to ambient temperature, the films were peeled from the substrates. The film thickness is approximately 30 µm.

### 4.2 Structural Characterization

Structural analysis was determined by XRD measurement (PANalytical B.V.), whereas the wavelength of the X‐ray was 1.542 Å (Cu Ka radiation, 40 kV, 40 mA). An electric field‐dependent XRD measurement was achieved by using the Trek 610E as the voltage supplier to generate electric fields onto the samples with electrodes (Quorum Q150RS Plus) on both sides. The connections between the sample and Trek were enabled by copper wires. To ensure the electrical insulation, Teflon film was fixed between the sample and sample holder. After the voltage was on, *θ*‐2*θ* scans were performed. FTIR spectra from 400 to 4000 cm^−1^ were measured by a Thermo Scientific Nicolet iS50R spectrometer in the attenuated total reflectance mode. XPS data were collected using a SCIENTIFIC ESCALAB 250Xi spectrometer (Thermo Scientific).

### 4.3 DSC Measurements

DSC measurements were performed using a Diamond DSC (PerkinElmer) under N_2_ atmosphere, with a temperature range from 30°C to 200°C and a heating rate of 10°C min^−1^.

### 4.4 Electrical Measurements

Before the electrical and electromechanical measurements, circular gold electrodes of a thickness of 50 nm were sputtered onto both sides of the films using a Quorum Q150RS Plus system. For dielectric and ferroelectric measurements, the area of the electrode was 12.56 mm^2^. Temperature‐dependent dielectric spectroscopy was measured using a Keysight E4980A LCR meter integrated with an oven, whereas a heating rate of 1°C min^−1^ was used. *P–E* loops were measured by a modified Sawyer‐Tower circuit driven by a Trek 610E high‐voltage amplifier.

### 4.5 Piezoelectric Measurements

Piezoelectric coefficient *d*_33_ was recorded using a quasi‐static *d*_33_ meter (PKD3‐2000, PolyK Technologies) through the Berlincourt method. In our *d*_33_ measurement, a varied static force from 0.3 N to 0.7 N was used. The results showed a slight decrease with increasing the magnitude of the static force (Figures S21 and S25). In this regard, *d*_33_ results measured by a static force of 0.5 N were used in this work, which is in line with recent *d*_33_ measurements on the soft polymer and nanocomposites [45, 46, 47, 48, 49]. Moreover, *d*_33_ value of commercial PVDF further verified the experimental setup used in this work [49]. The size of samples was 1 cm^2^. Electric‐field‐induced strain measurement was used to determine converse *d*_33_ through the converse piezoelectric effect, where the samples underwent a deformation in response to an electric field. Strain was recorded by a laser interferometer (SIOS SP‐S 120E). The electric field with a triangular bipolar wave at 1 Hz was used. The area of the electrode was 28.26 mm^2^. Before *d*_33_ and strain measurements, the polymer films were polarized by using contact poling at room temperature (10 mins at a DC electric field of 150 MV m^−1^). For *d*_33_ measurement under electrical cycles, an electric field of 15 MV m^−1^ was applied (10 Hz). The cyclic mechanical force (3 Hz) was applied through a pressure‐controlled motor (PR‐BDM8‐100F, PURI Materials) at room temperature. The converse *d*_33_ measurement was maintained for temperature rising to 65°C.

### 4.6 DFT Calculations

DFT calculations were carried out using the Vienna Ab initio Simulation Package (VASP) [50, 51]. The electron‐ion interactions were described by the projector augmented wave (PAW) method [52, 53]. The exchange‐correlation functional was treated within the generalized gradient approximation (GGA) in the Perdew–Burke–Ernzerhof (PBE) form [54]. A plane‐wave basis set with a kinetic energy cutoff of 400 eV was employed. The electronic self‐consistent iteration convergence was set to 10^−5^ eV. For geometry optimization, the Hellmann–Feynman forces on all atoms were relaxed to less than 0.02 eV Å^−1^. The Brillouin zone sampling was performed using the Γ‐point for k‐space integration [55].

We trained the *α* phase through convergence test under different conditions (i.e., kpoints, energy cutoffs, electronic self‐consistent iteration convergence, and dispersion corrections). In our test, we changed one single parameter presented in condition 1 (1×1×1 kpoints, energy cutoff of 400 eV, electronic self‐consistent iteration convergence of 10^−5^ eV without dispersion corrections) while fixing the remaining other parameters and performed the structural relaxations. The detailed energy values under varied conditions *U* remained nearly unchanged with a negligible variation rate defined as δ*U*/*U*, as summarized in Table S1. We also compared the results under different sets of convergence parameters (i.e., 1×1×1 kpoints, energy cutoff of 400 eV electronic self‐consistent iteration convergence of 10^−5^ eV without dispersion corrections versus 1×4×4 kpoints, energy cutoff of 500 eV electronic self‐consistent iteration convergence of 10^−6^ eV with dispersion corrections). The results showed that the difference in energy only yielded a small variation rate. Therefore, 1×1×1 kpoints and an energy cutoff of 400 eV without dispersion corrections were used with the electronic self‐consistent iteration convergence of 10^−5^ eV. The models on PVDF with different chain conformations were built based on well‐established structures reported in the literature [56, 57]. Four distinct crystalline phases were investigated including the *α*, *β*, *δ*, and 3/1‐helical phases. Each system was modeled under periodic boundary conditions with two PVDF chains containing 20 CF_2_CH_2_ units in a simulation box. 5 mol% HH defect, 5 mol% HHTT, and grafting with butylamine were included in our calculations.

## Conflicts of Interest

The authors declare no conflicts of interest.

## Supporting information




**Supporting File**: advs74900‐sup‐0001‐SuppMat.pdf.

## Data Availability

The data that support the findings of this study are available from the corresponding author upon reasonable request.
