# Supplementary material for: Morphotropic Phase Boundary in Graft Poly(Vinylidene Fluoride‐co‐Tetrafluoroethylene) With High Curie Temperature
Source: Adv Sci (Weinh). 2026 Mar 19;13(31):e74900. doi: 10.1002/advs.74900 (PMC13587932; doi:10.1002/advs.74900)
Supplement: Supplementary file 1 [file ADVS-13-e74900-s001.pdf]

Supporting Information

Morphotropic phase boundary in graft poly(vinylidene fluoride-*co*-tetrafluoroethylene) with high Curie temperature

Zekai Fei#, Chenyi Li#, Yuquan Liu, Yang Li, Yun Zhang*, Huamin Zhou*, Yang Liu**

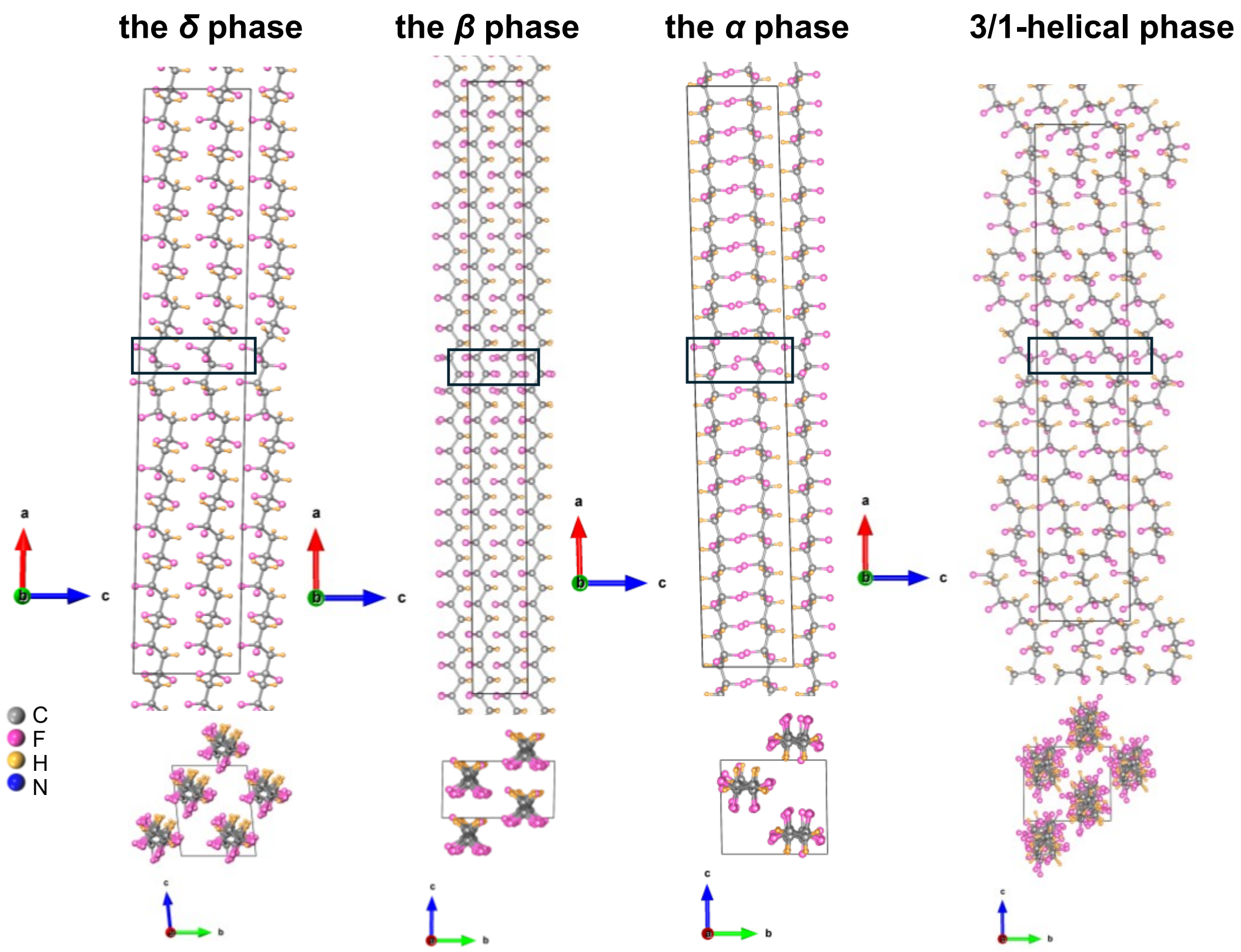

Figure S1. Pristine P(VDF-TFE) of different chain conformations without HHTT defects. The black boxes indicate the HH defects.

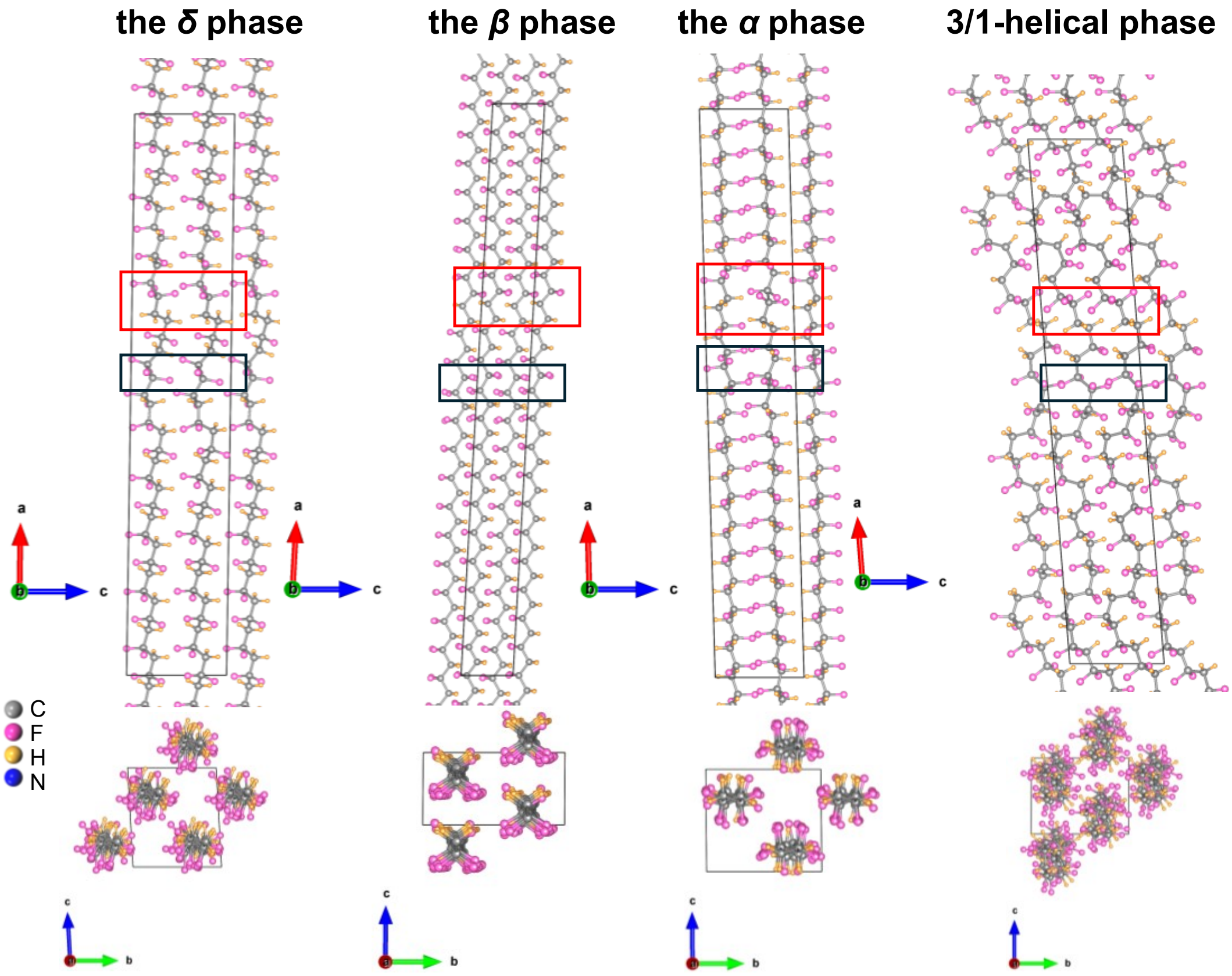

Figure S2. Pristine P(VDF-TFE) of different chain conformations with HHTT defects. The black boxes indicate the HH defects. The red boxes indicate the HHTT defects.

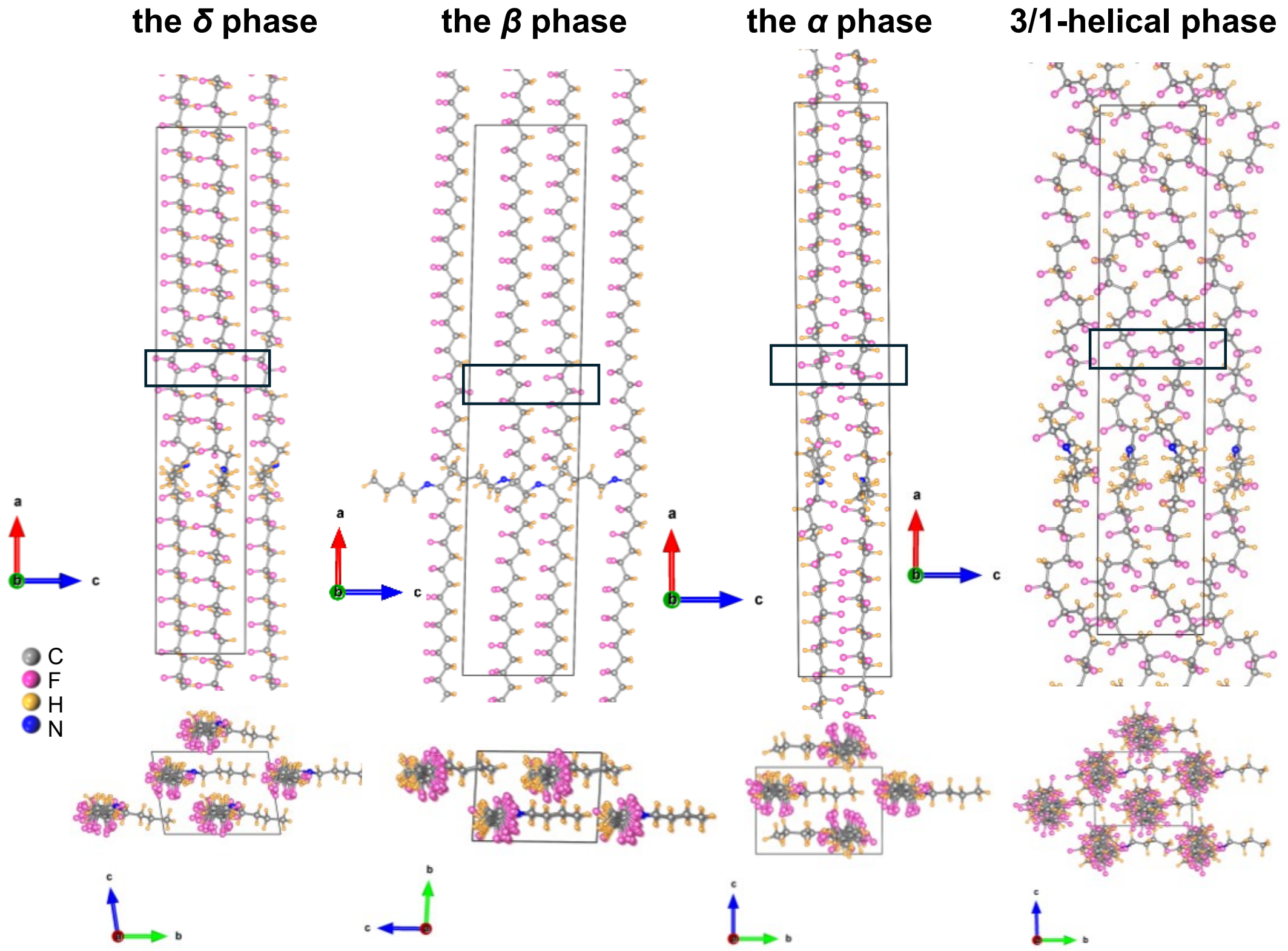

Figure S3. Graft P(VDF-TFE) of different chain conformations without HHTT defects. The black boxes indicate the HH defects.

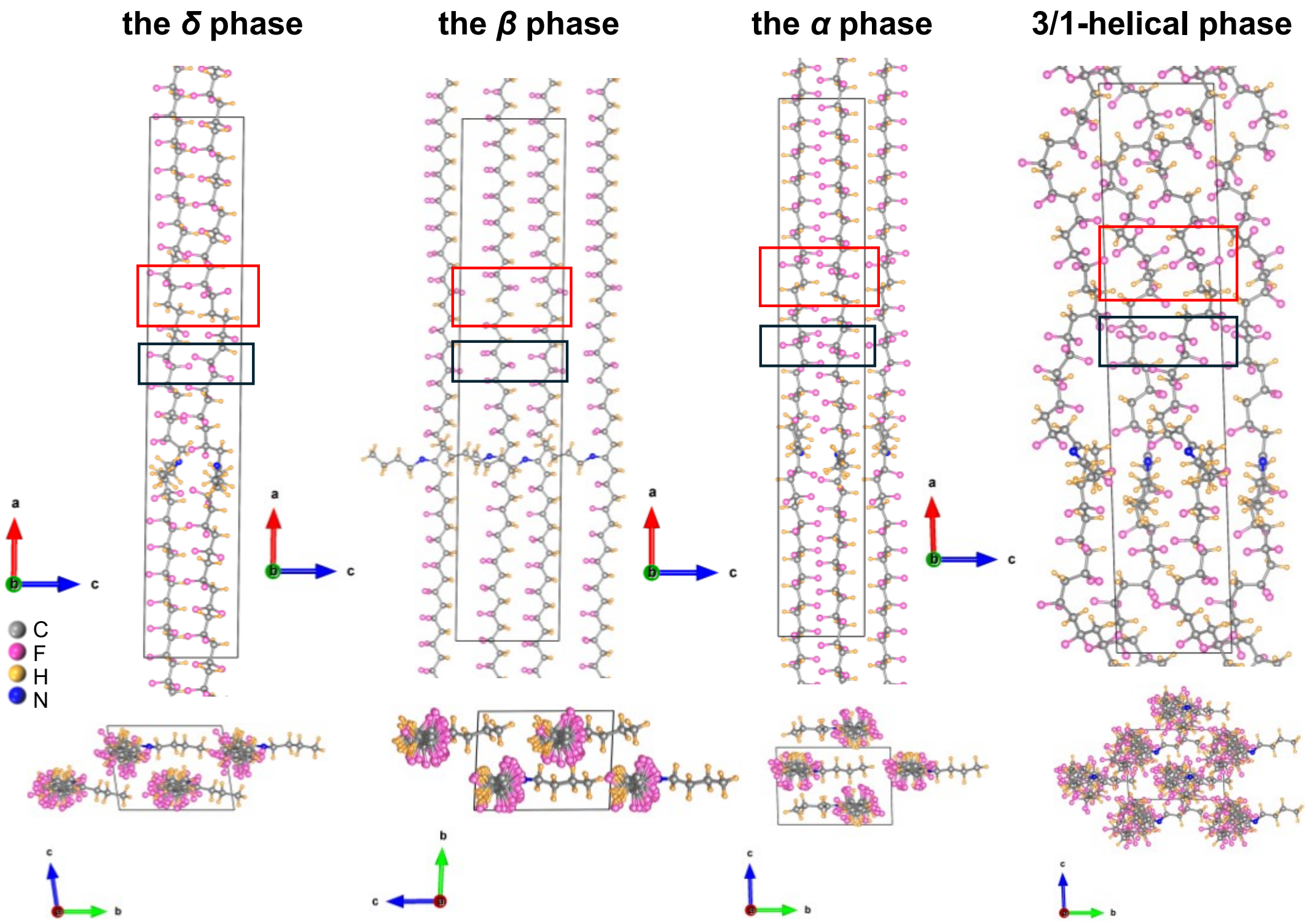

Figure S4. Graft P(VDF-TFE) of different chain conformations with HHTT defects. The black boxes indicate the HH defects. The red boxes indicate the HHTT defects.

a

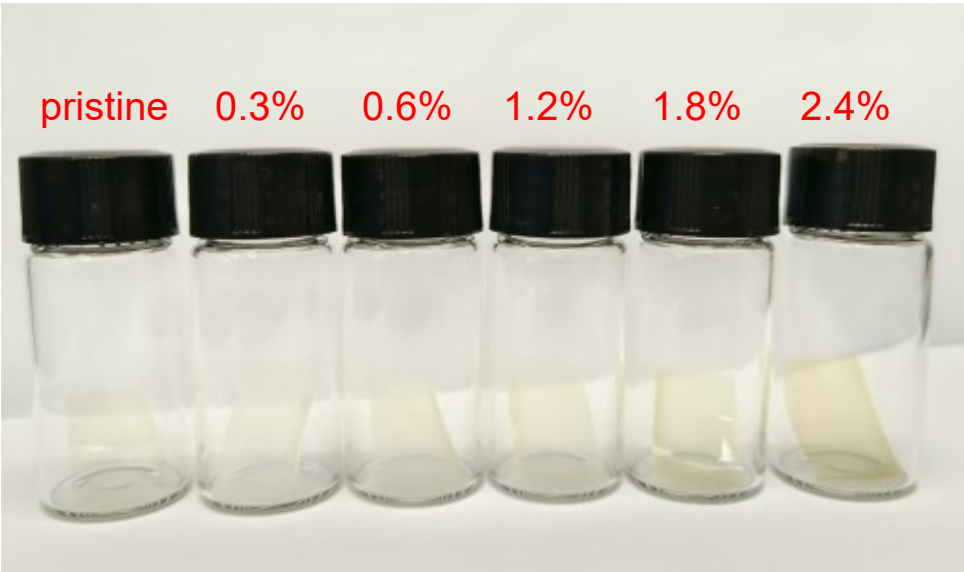

b

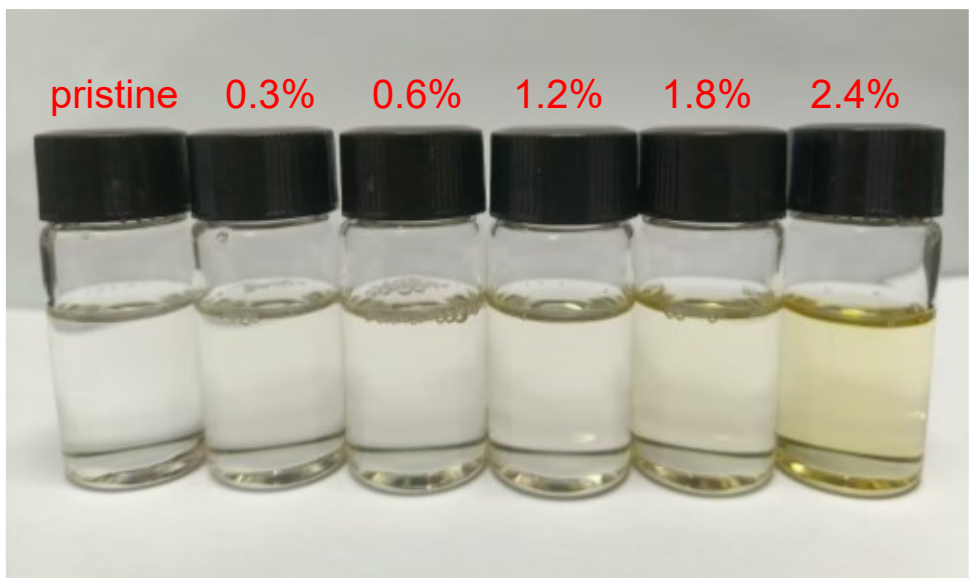

Figure S5. Photo images before (a) and after (b) dissolving of graft polymer films into N,N-Dimethylformamide.

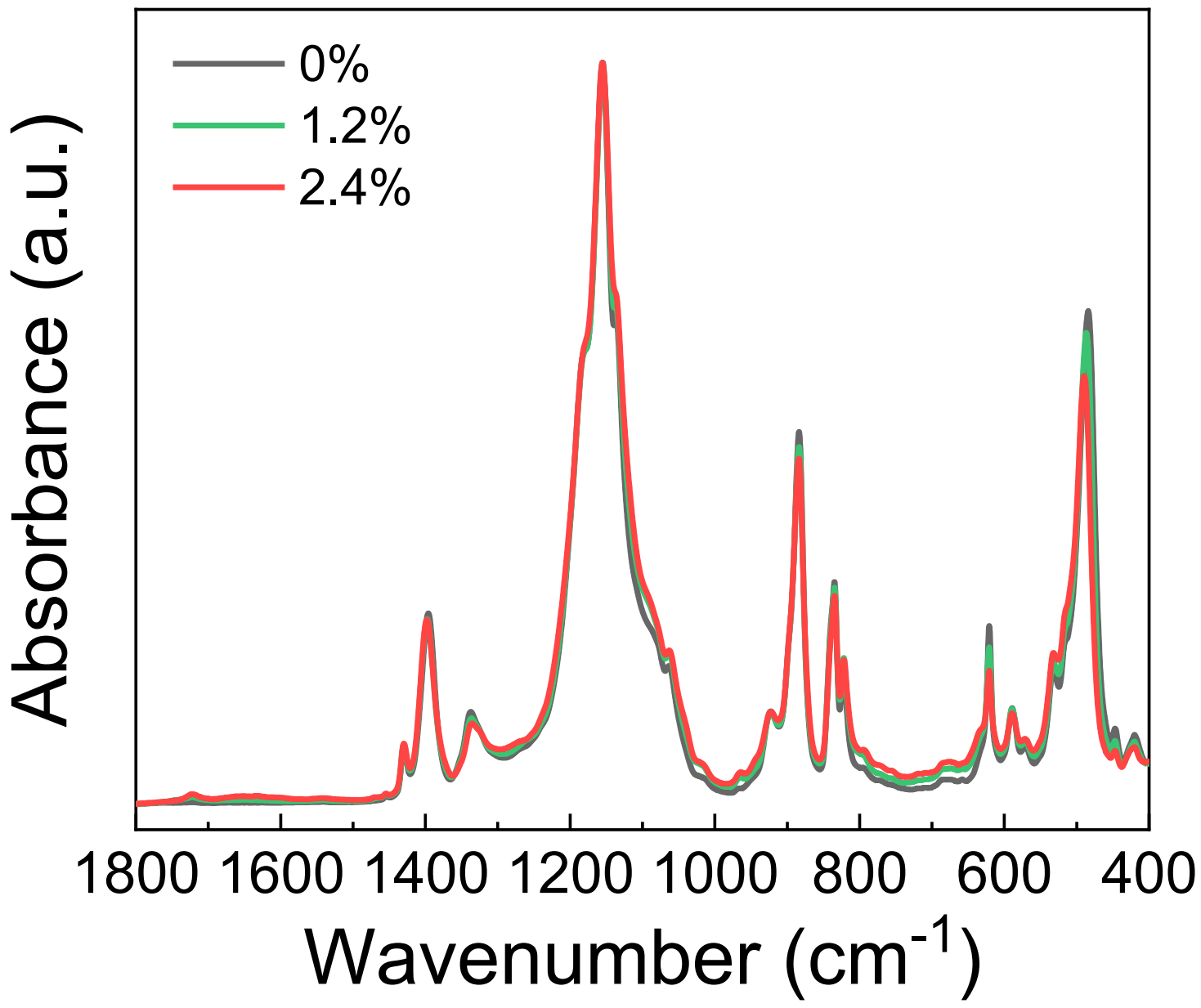

Figure S6. FTIR spectra with various grafting densities.

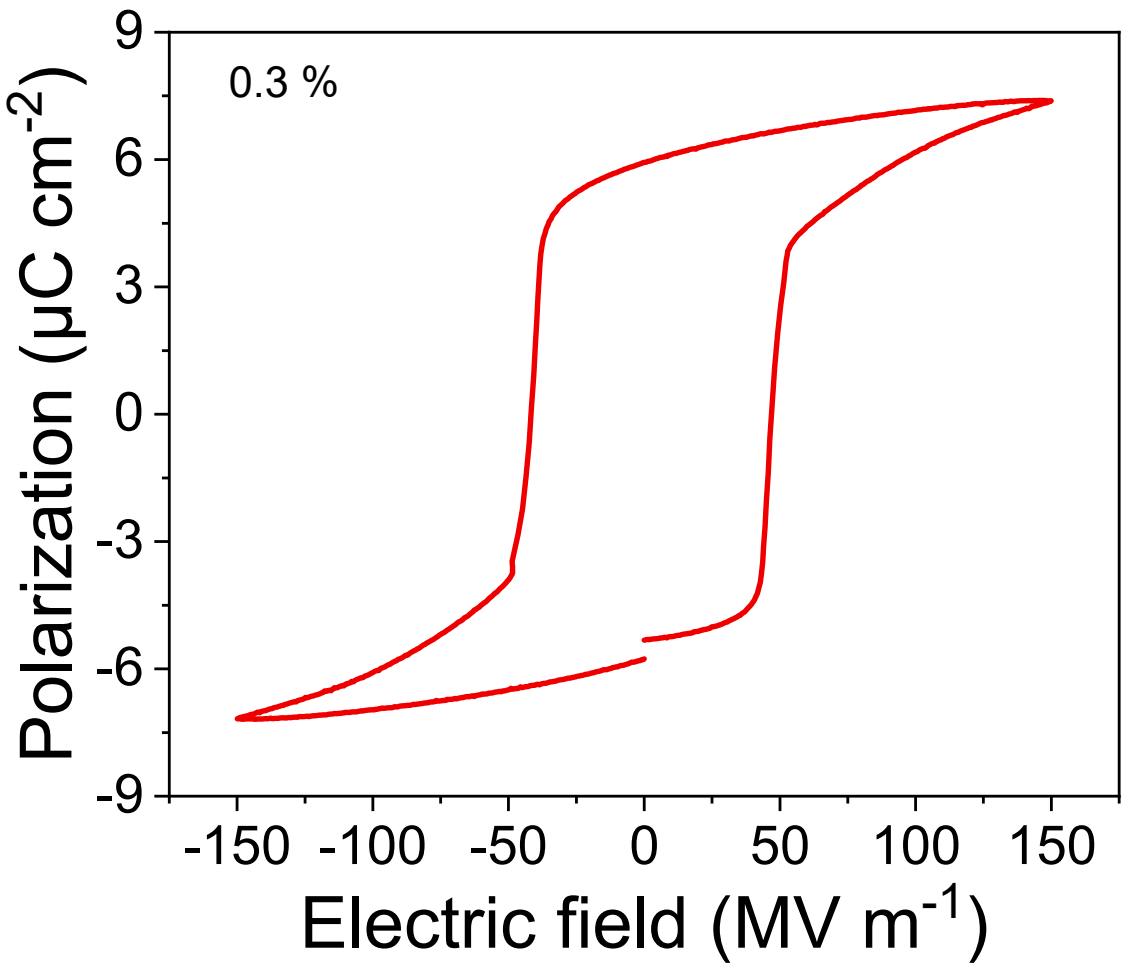

Figure S7. *P-E* loop obtained in graft P(VDF-TFE) with a grafting density of 0.3%.

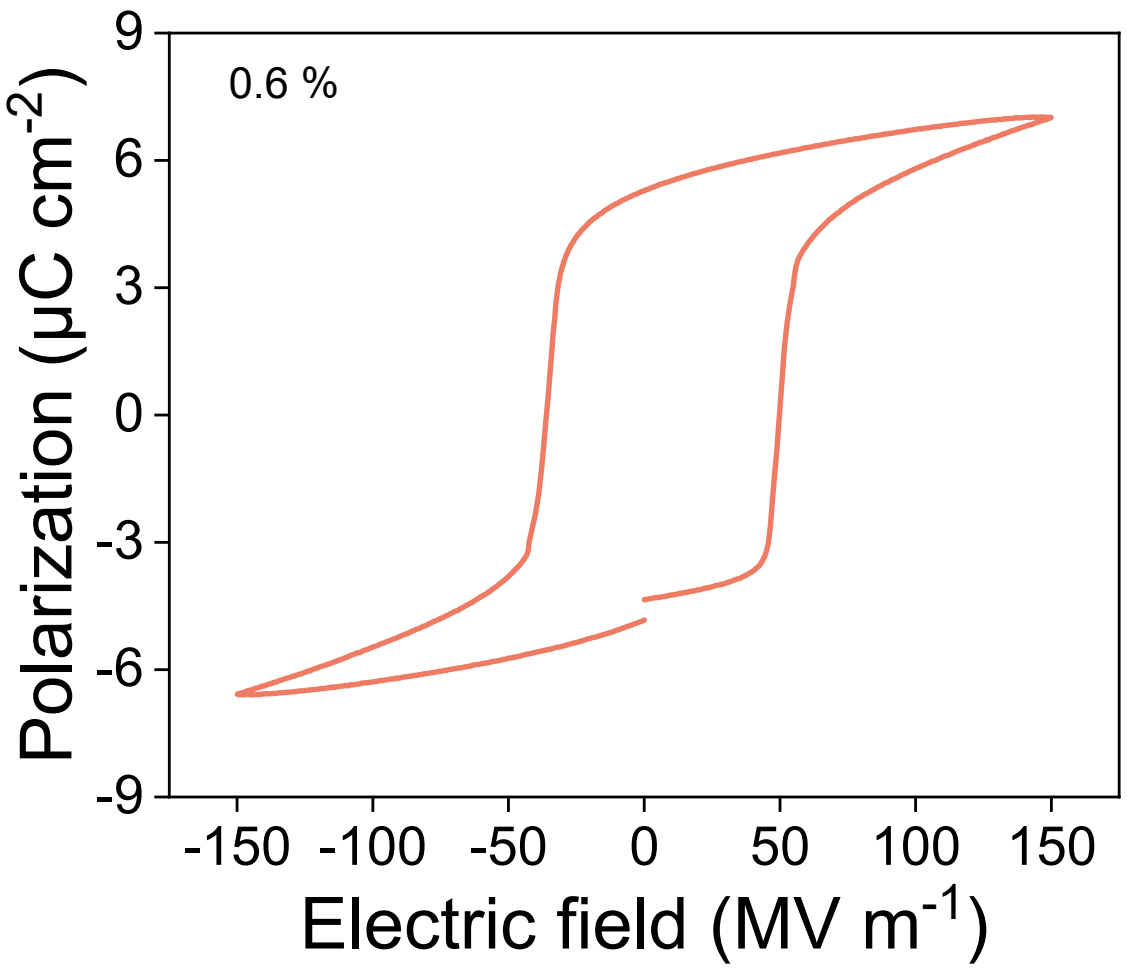

Figure S8. *P*-*E* loop obtained in graft P(VDF-TFE) with a grafting density of 0.6%.

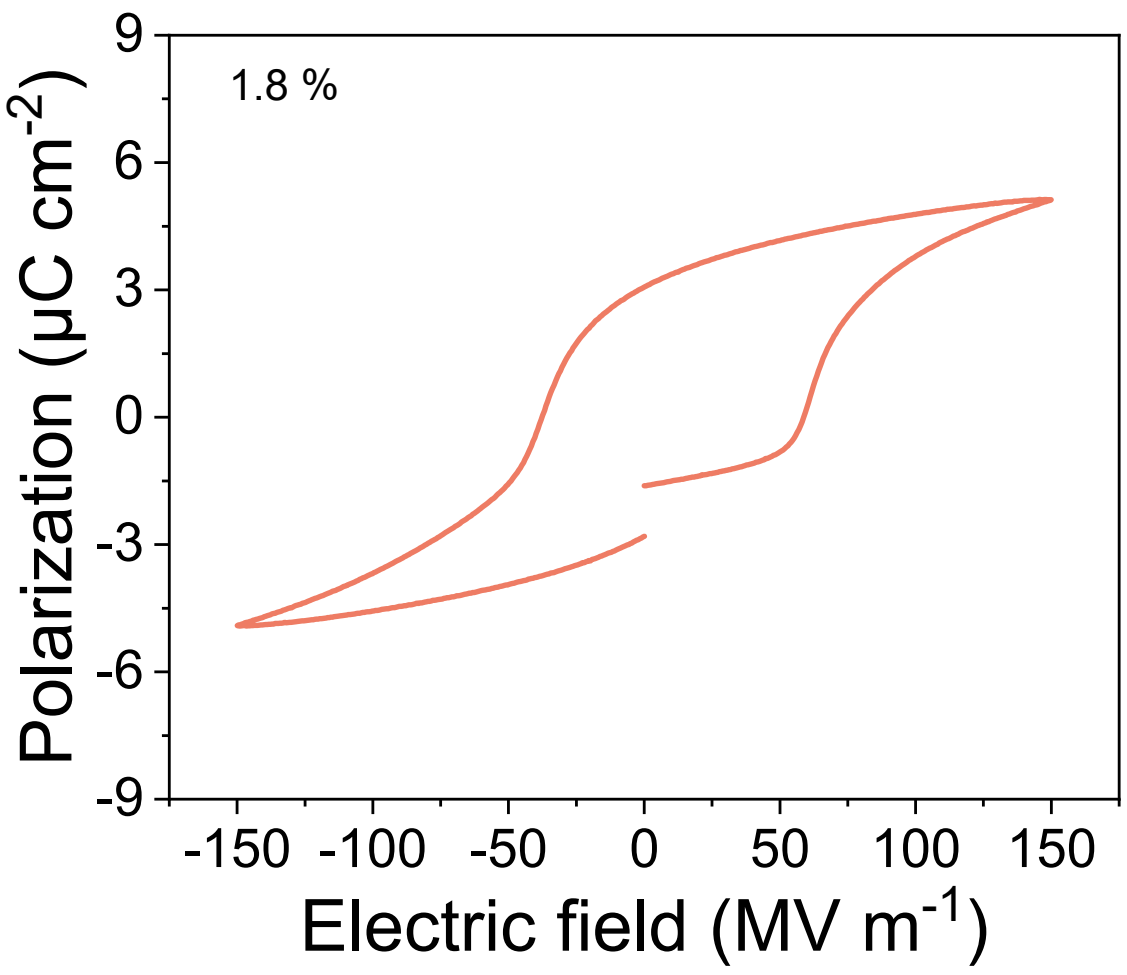

Figure S9. *P-E* loop obtained in graft P(VDF-TFE) with a grafting density of 1.8%.

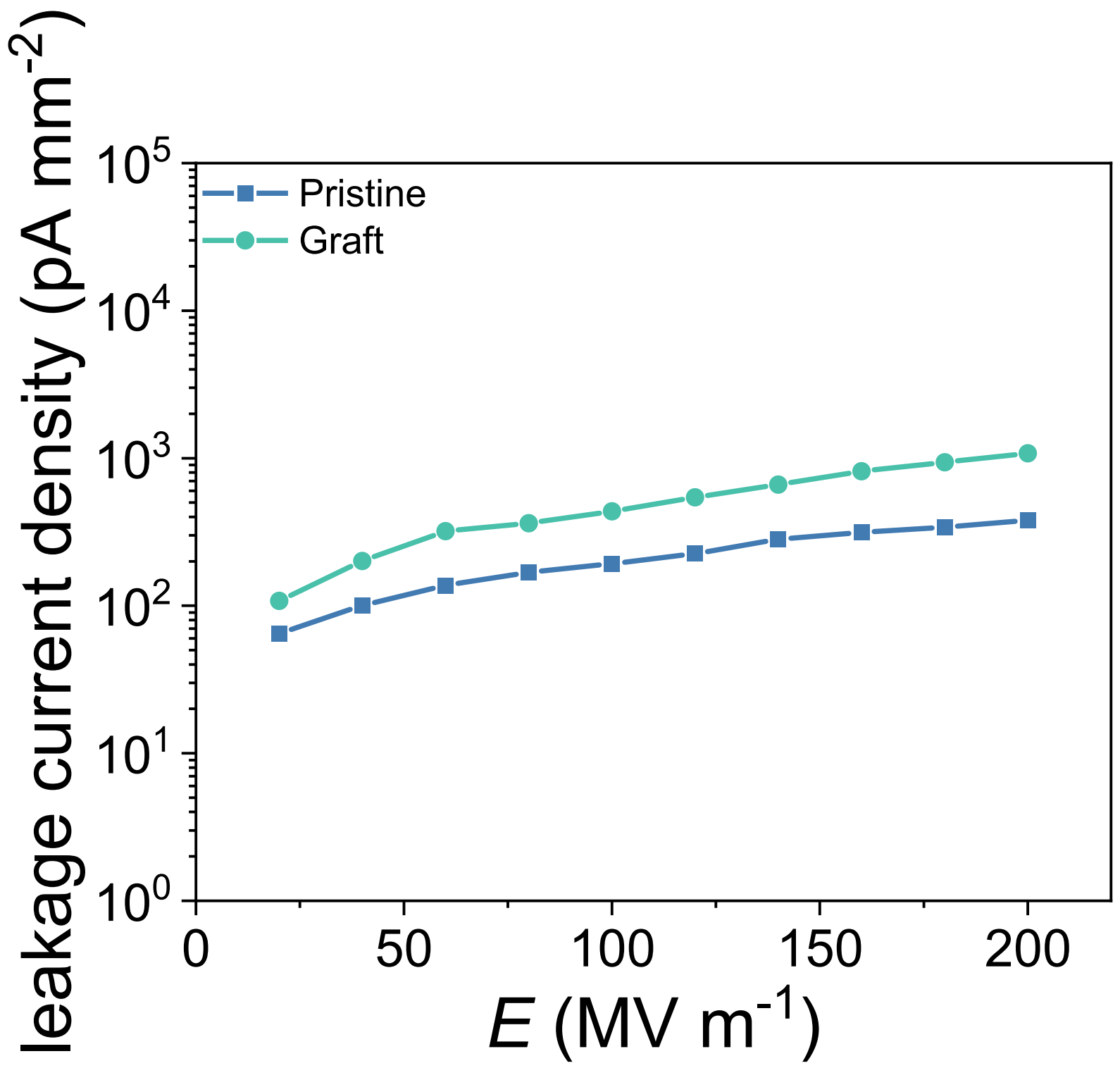

Figure S10. Leakage current density as a function of electric field in pristine and graft P(VDF-TFE).

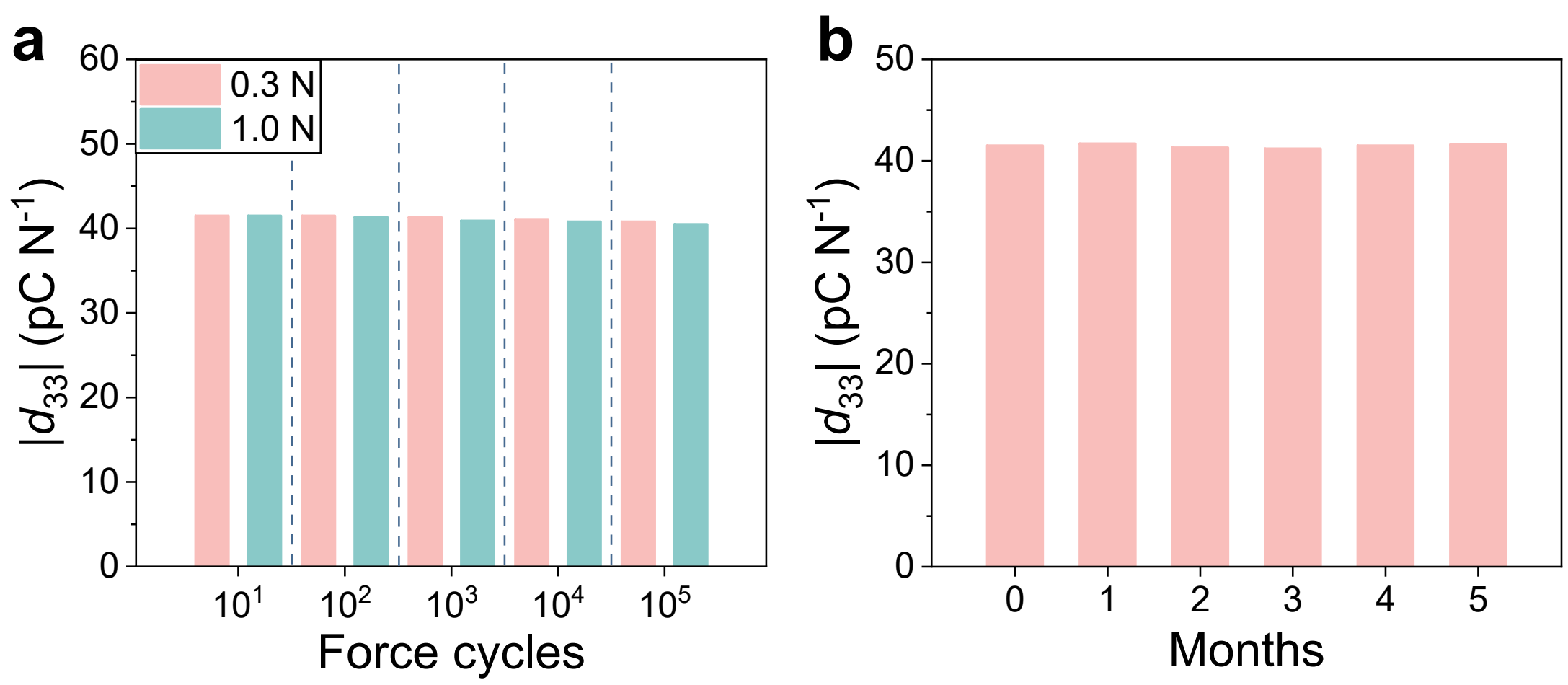

Figure S11. (a) d_{33} as a function of mechanical cycles with a force of 0.3 and 1 N (3 Hz). (b) Stability of d_{33} a function of time.

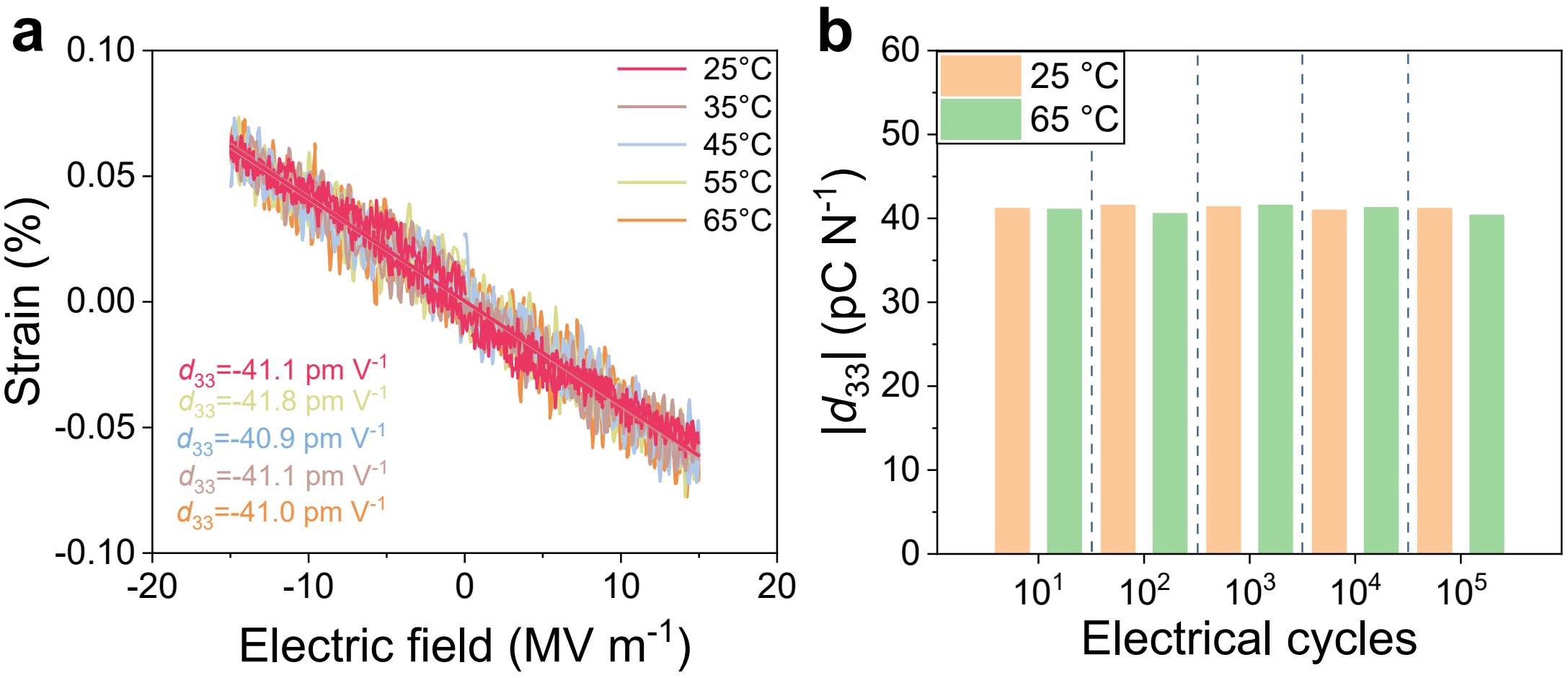

Figure S12. (a) Electric field-induced strain under different temperatures. (b) Stability of d_{33} a function of electrical cycles under different temperatures (25 MV m^{-1}).

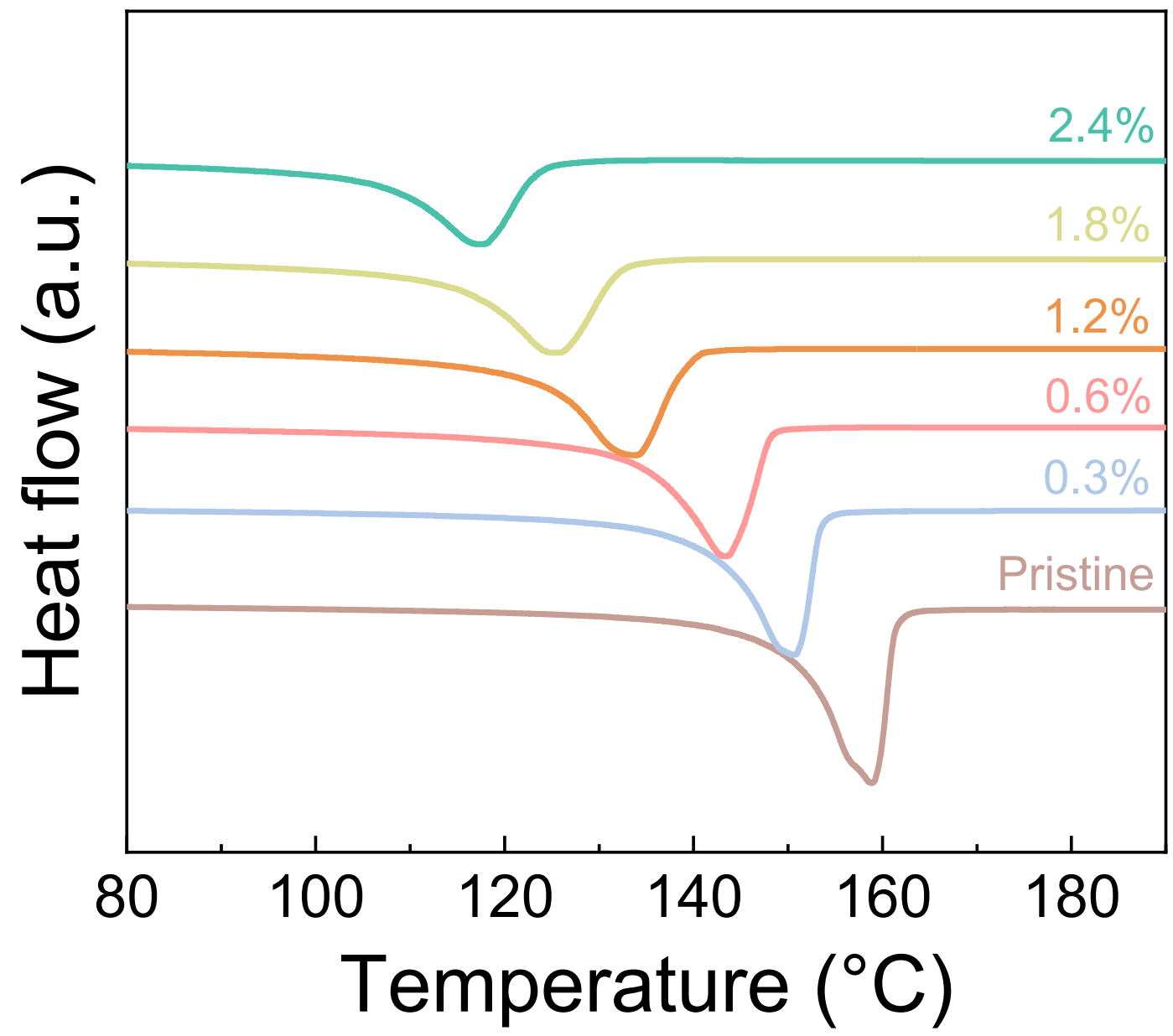

Figure S13. DSC heating scans in graft P(VDF-TFE) with various grafting densities.

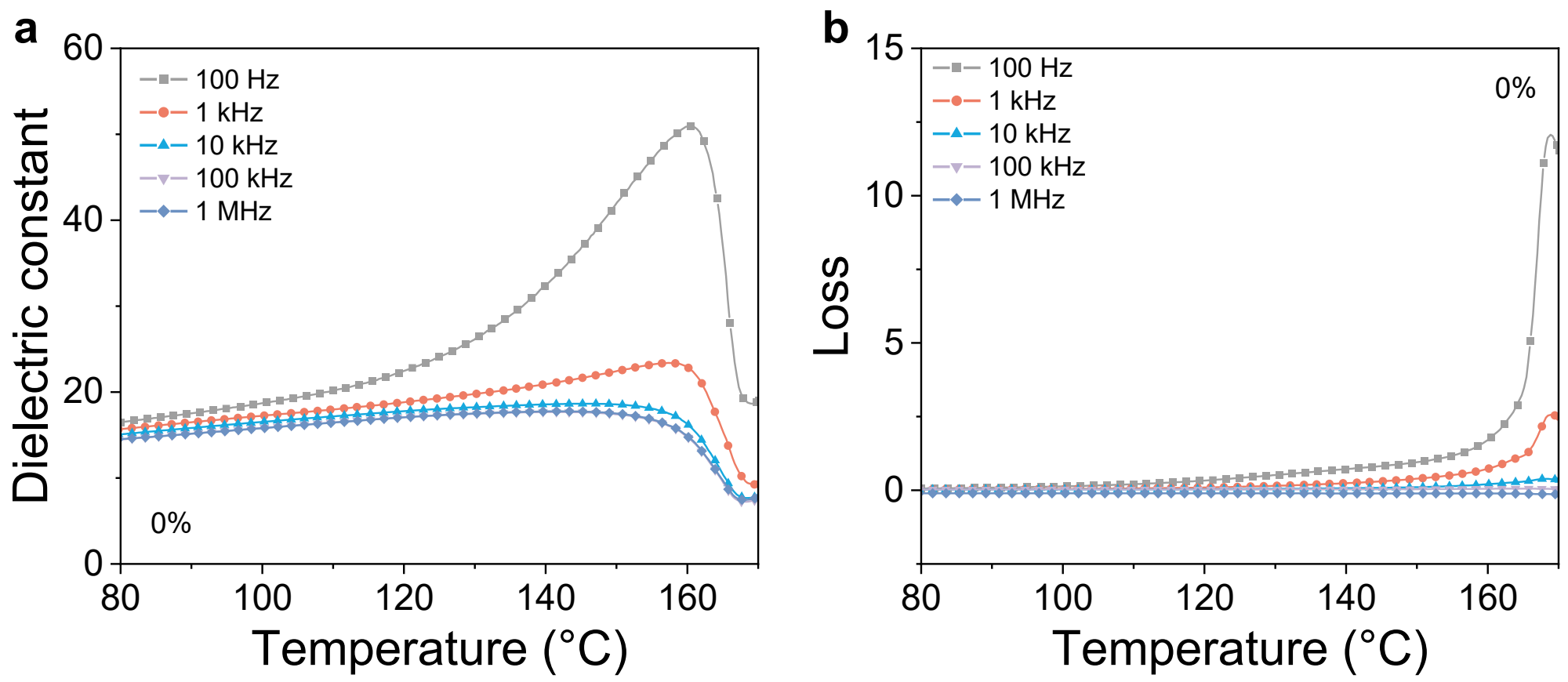

Figure S14. Temperature-dependent dielectric constant (a) and loss (b) in pristine P(VDF-TFE).

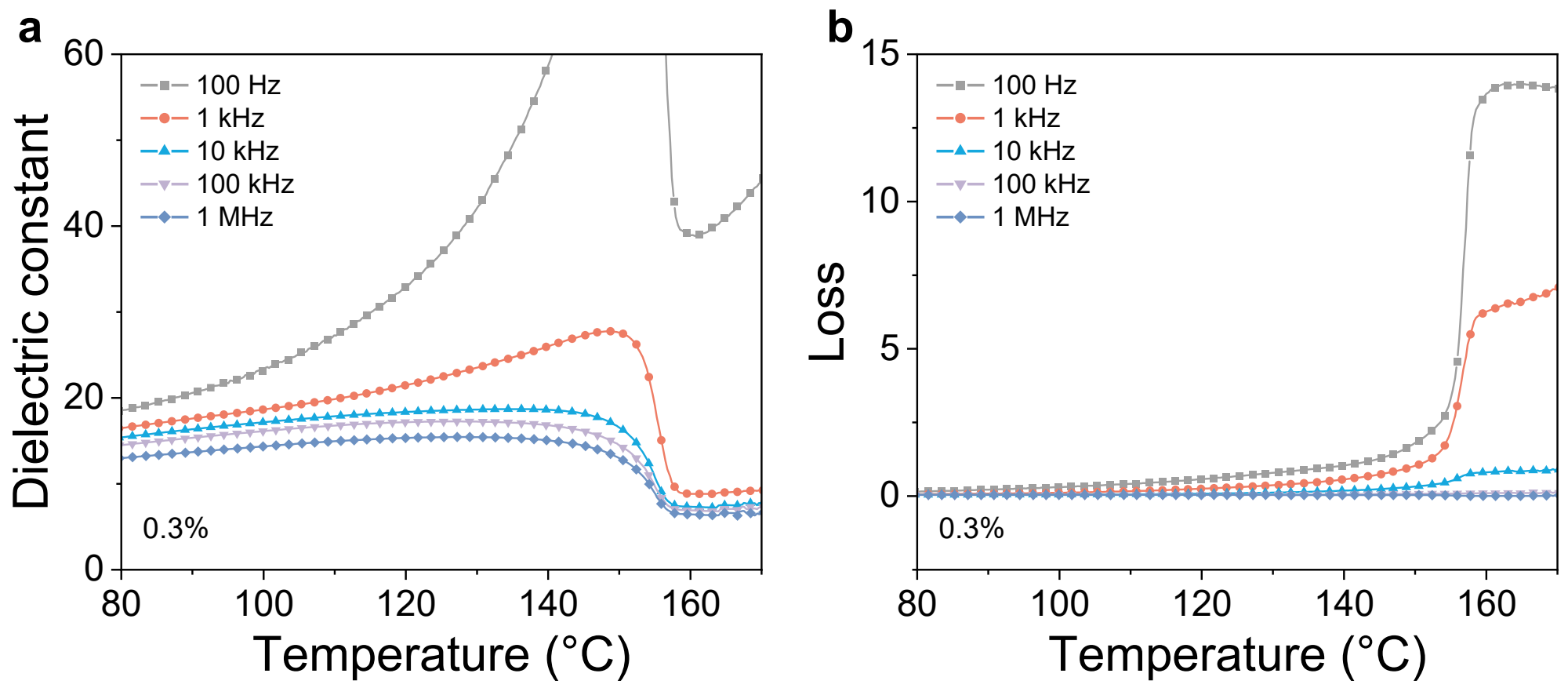

Figure S15. Temperature-dependent dielectric constant (a) and loss (b) in graft P(VDF-TFE) with a grafting density of 0.3%.

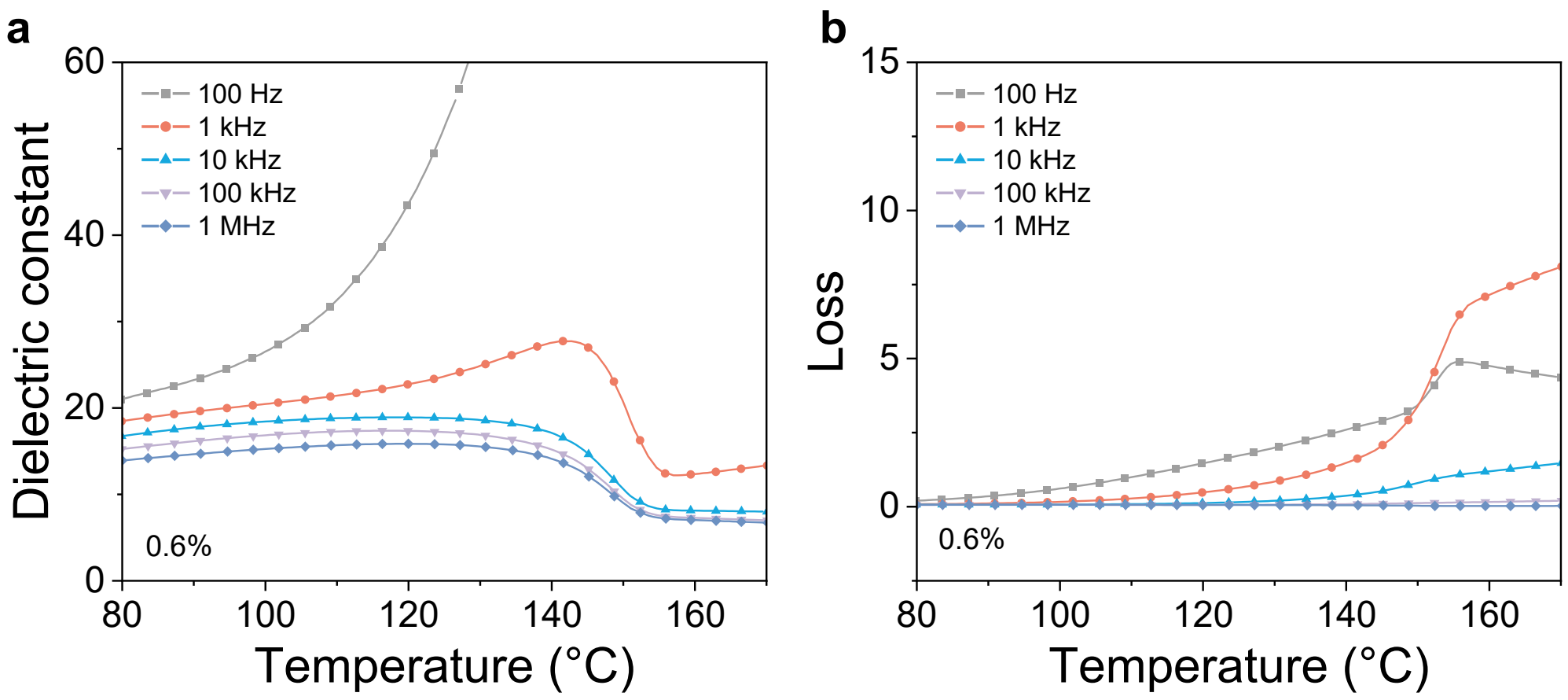

Figure S16. Temperature-dependent dielectric constant (a) and loss (b) in graft P(VDF-TFE) with a grafting density of 0.6%.

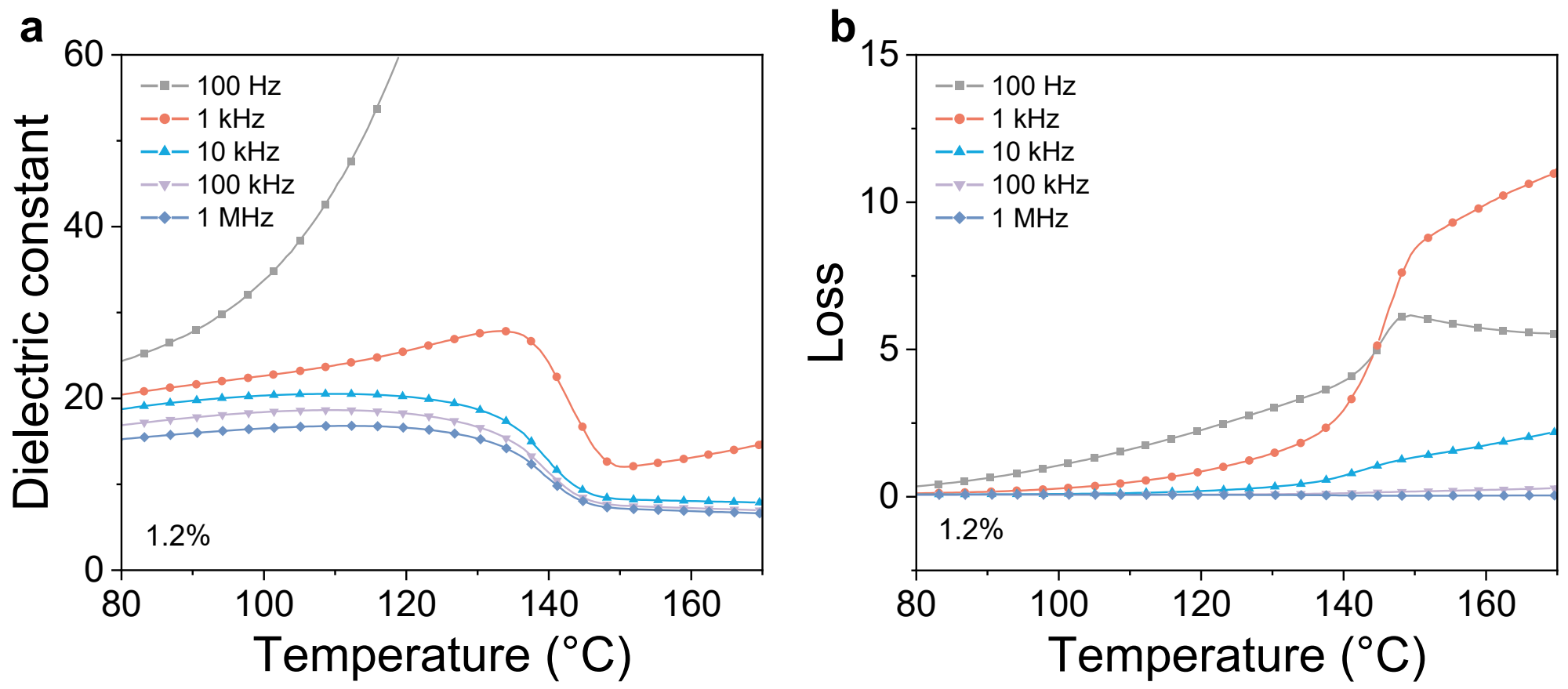

Figure S17. Temperature-dependent dielectric constant (a) and loss (b) in graft P(VDF-TFE) with a grafting density of 1.2%.

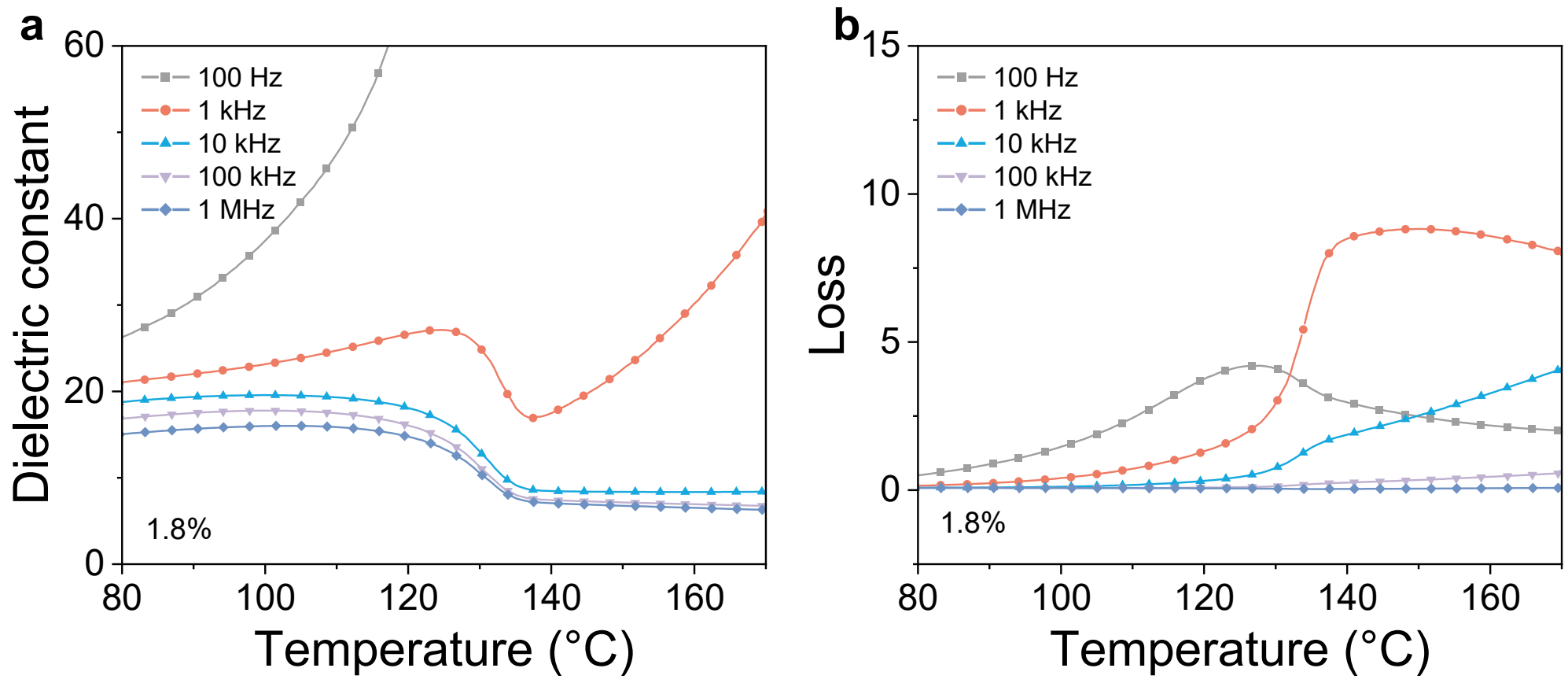

Figure S18. Temperature-dependent dielectric constant (a) and loss (b) in graft P(VDF-TFE) with a grafting density of 1.8%.

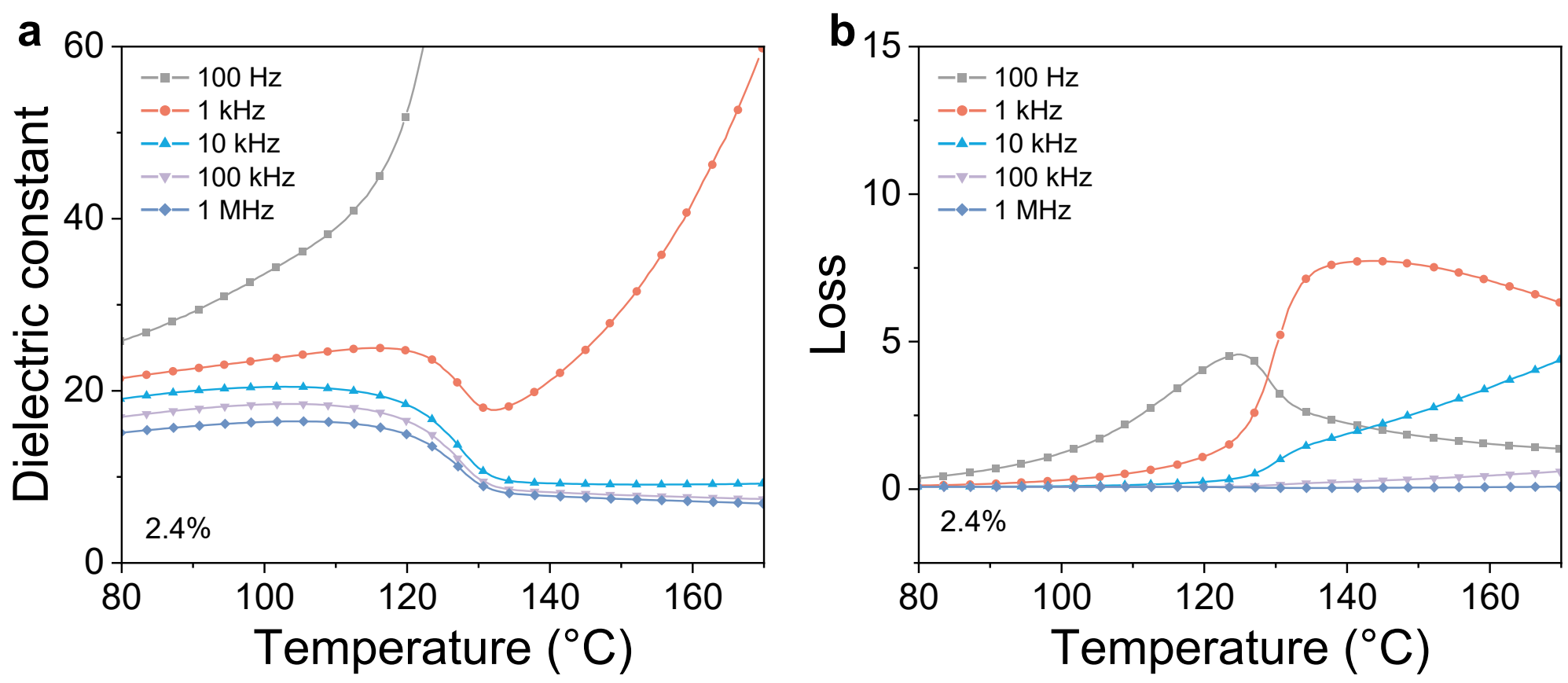

Figure S19. Temperature-dependent dielectric constant (a) and loss (b) in graft P(VDF-TFE) with a grafting density of 2.4%.

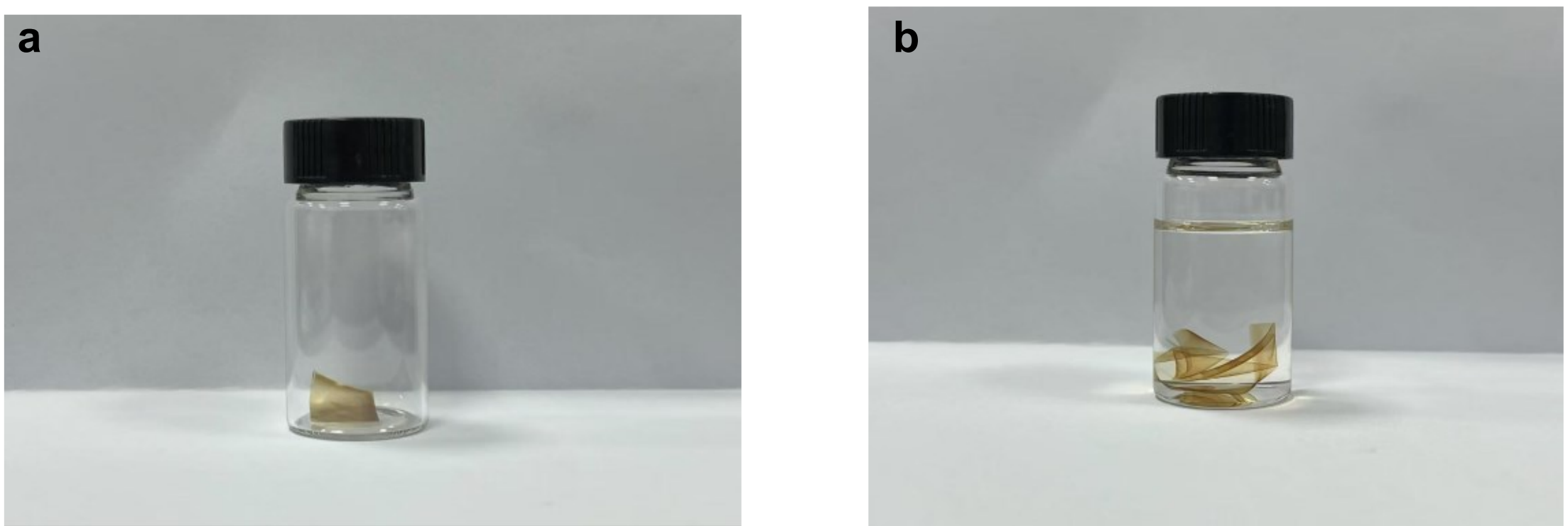

Figure S20. Photo images before (a) and after (b) dissolving of graft polymer films into N,N-Dimethylformamide with a grafting density of 3%.

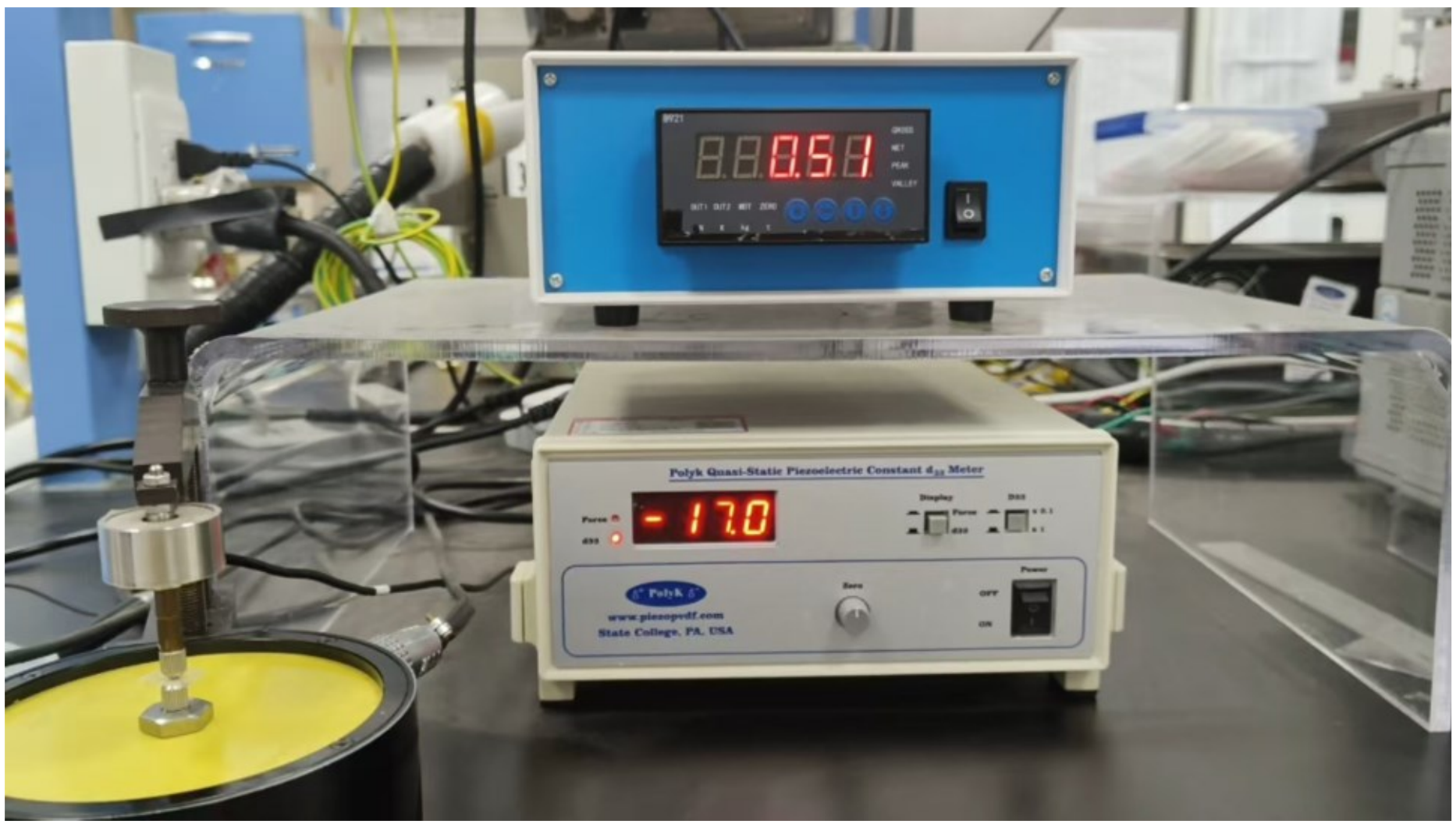

Figure S21. Photo image of d_{33} result measured by d_{33} meter in pristine P(VDF-TFE).

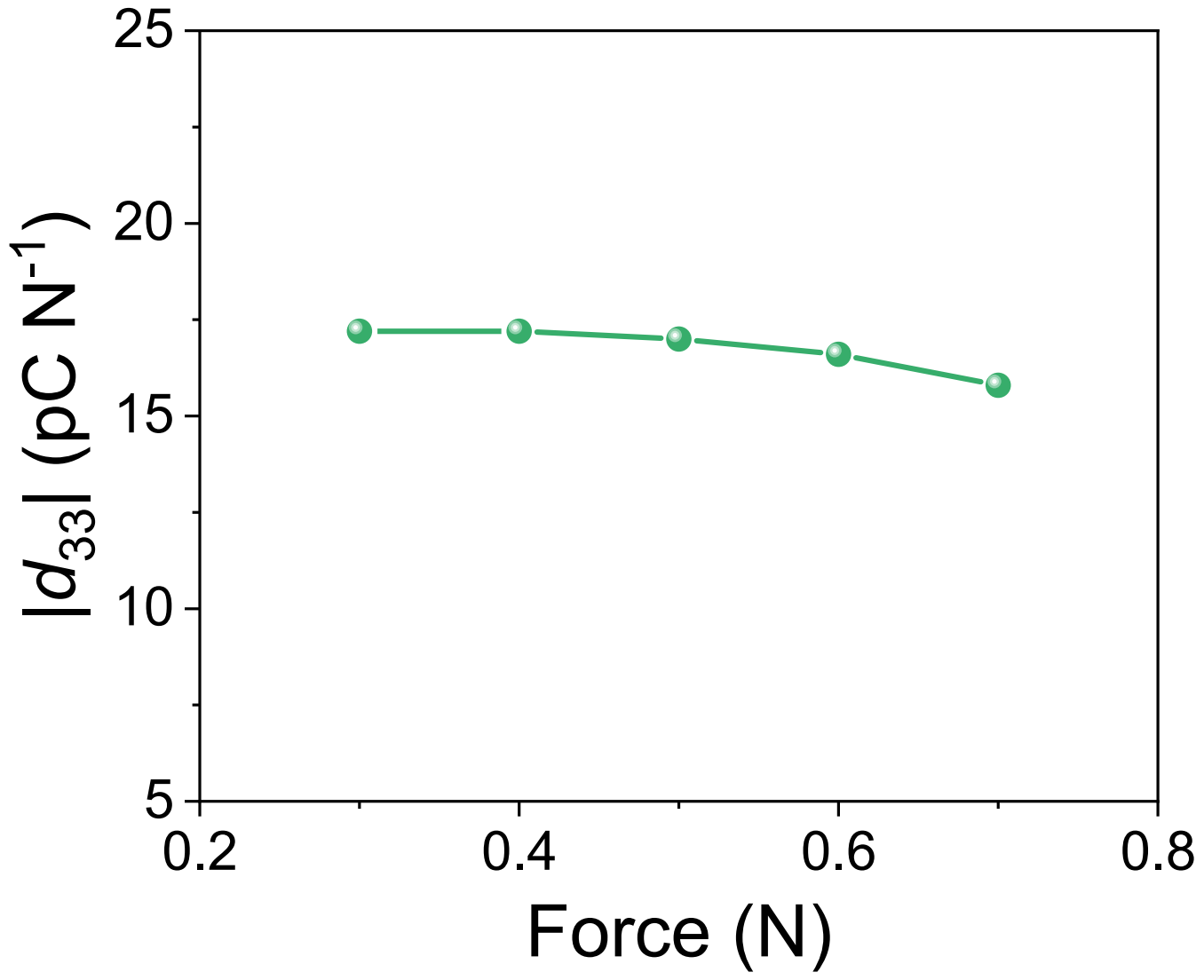

Figure S22. d_{33} measured under different static forces by d_{33} meter (pristine P(VDF-TFE)).

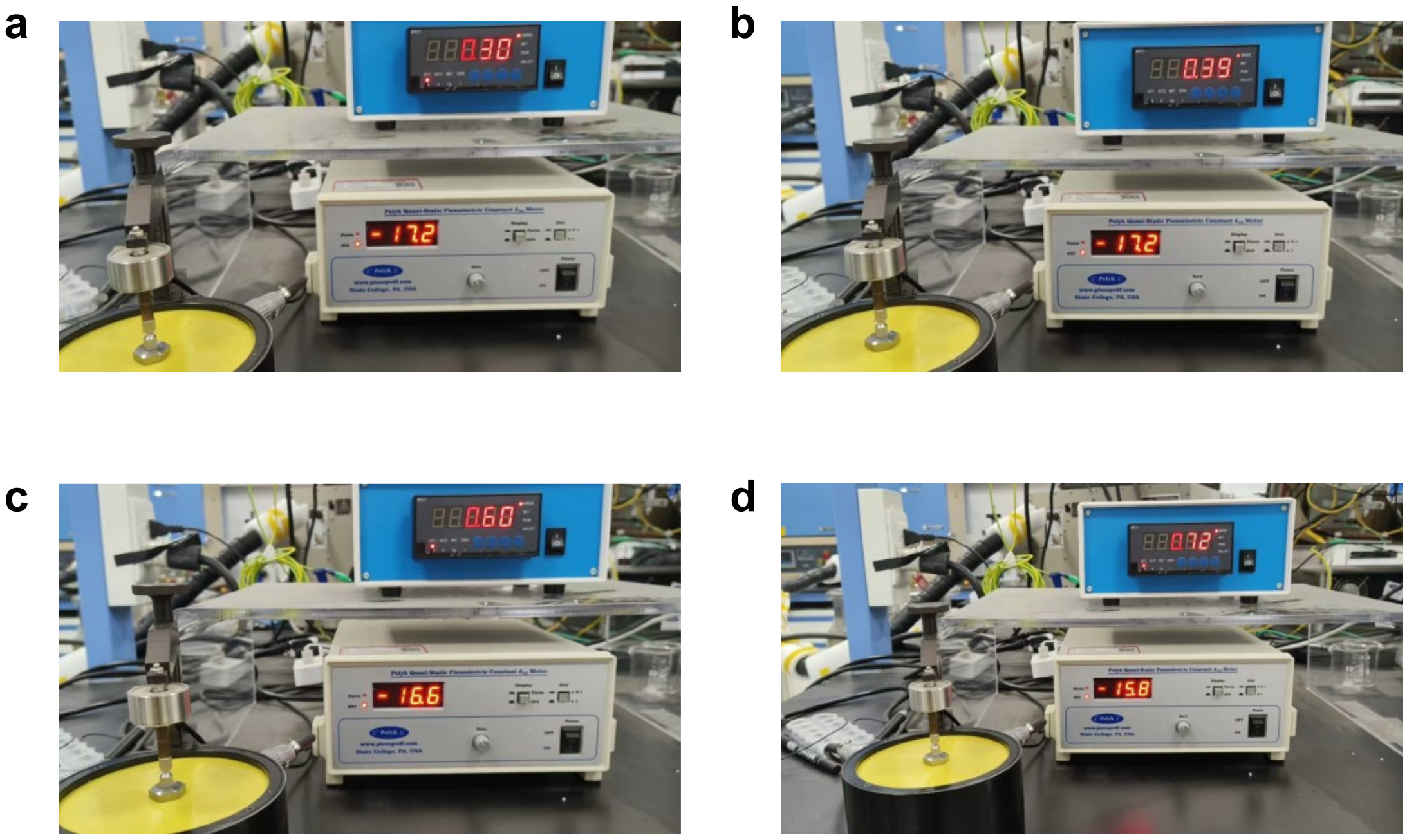

Figure S23. Photo images of d_{33} results measured under different static forces by d_{33} meter. (a) 0.30 N. (b) 0.39 N. (c) 0.60 N. (d) 0.72 N. (pristine P(VDF-TFE))

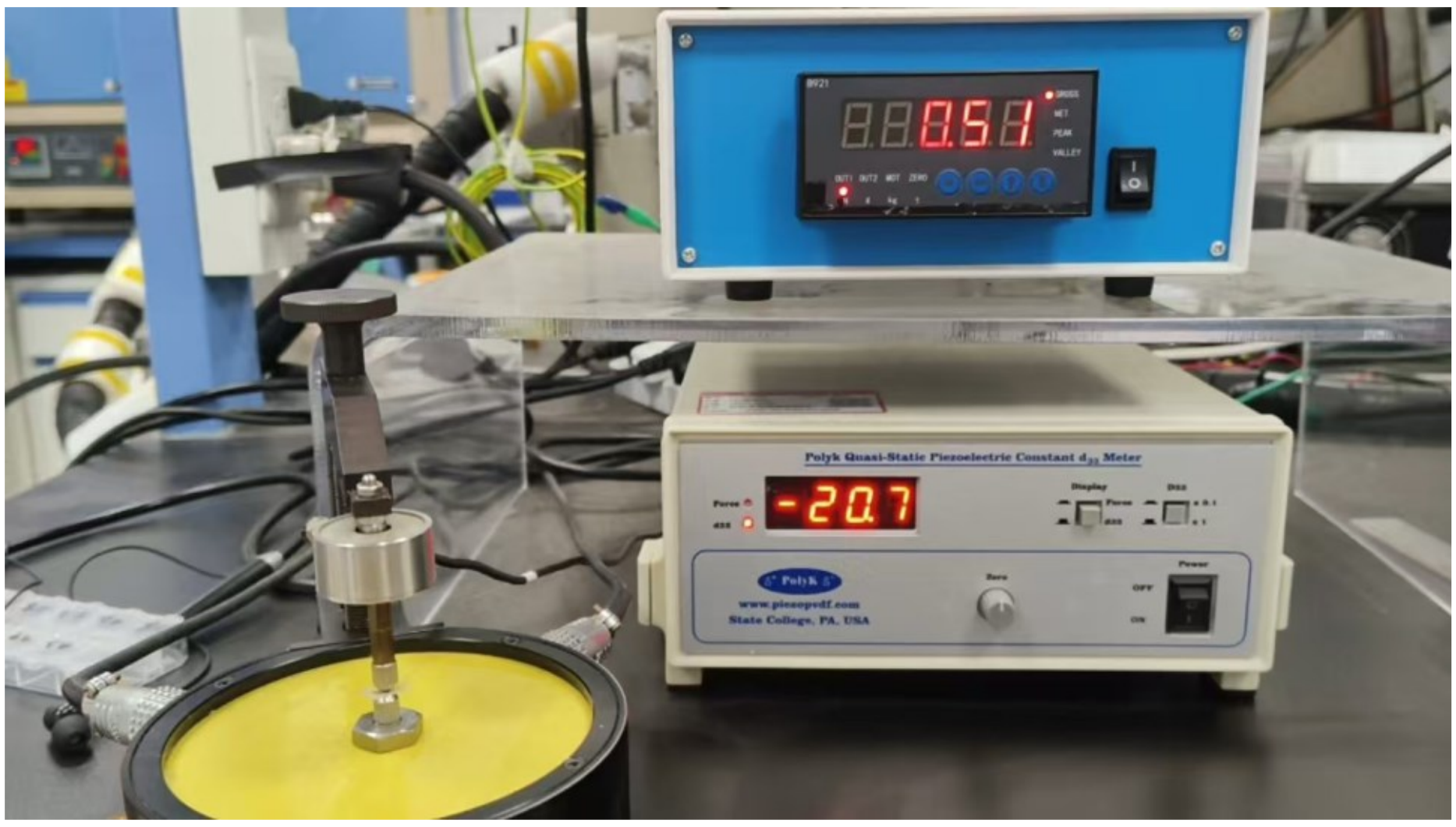

Figure S24. Photo image of d_{33} result measured by d_{33} meter in pristine P(VDF-TrFE) 80/20 mol%.

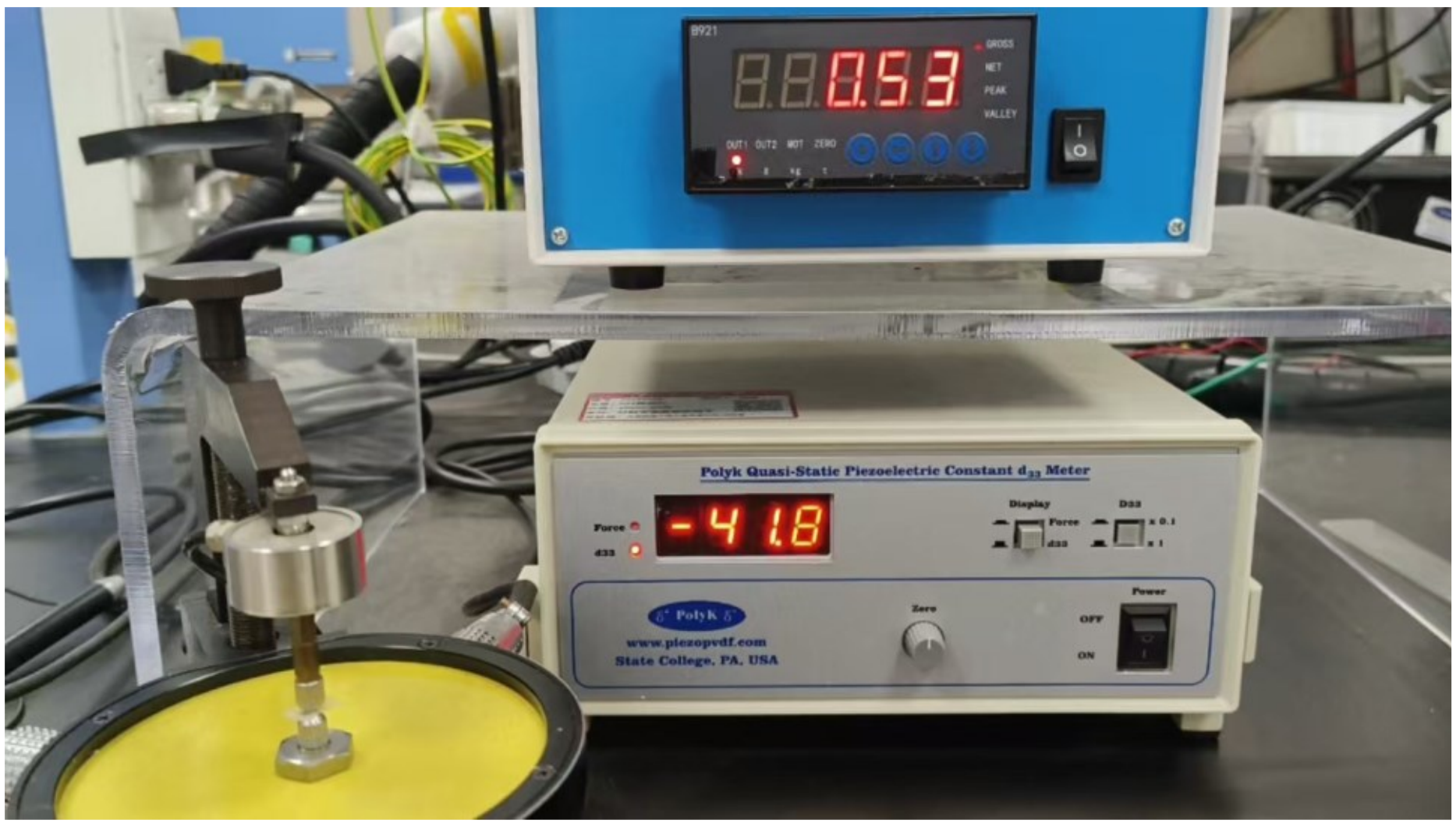

Figure S25. Photo image of d_{33} result measured by d_{33} meter in graft P(VDF-TFE) with a grafting density of 1.2%.

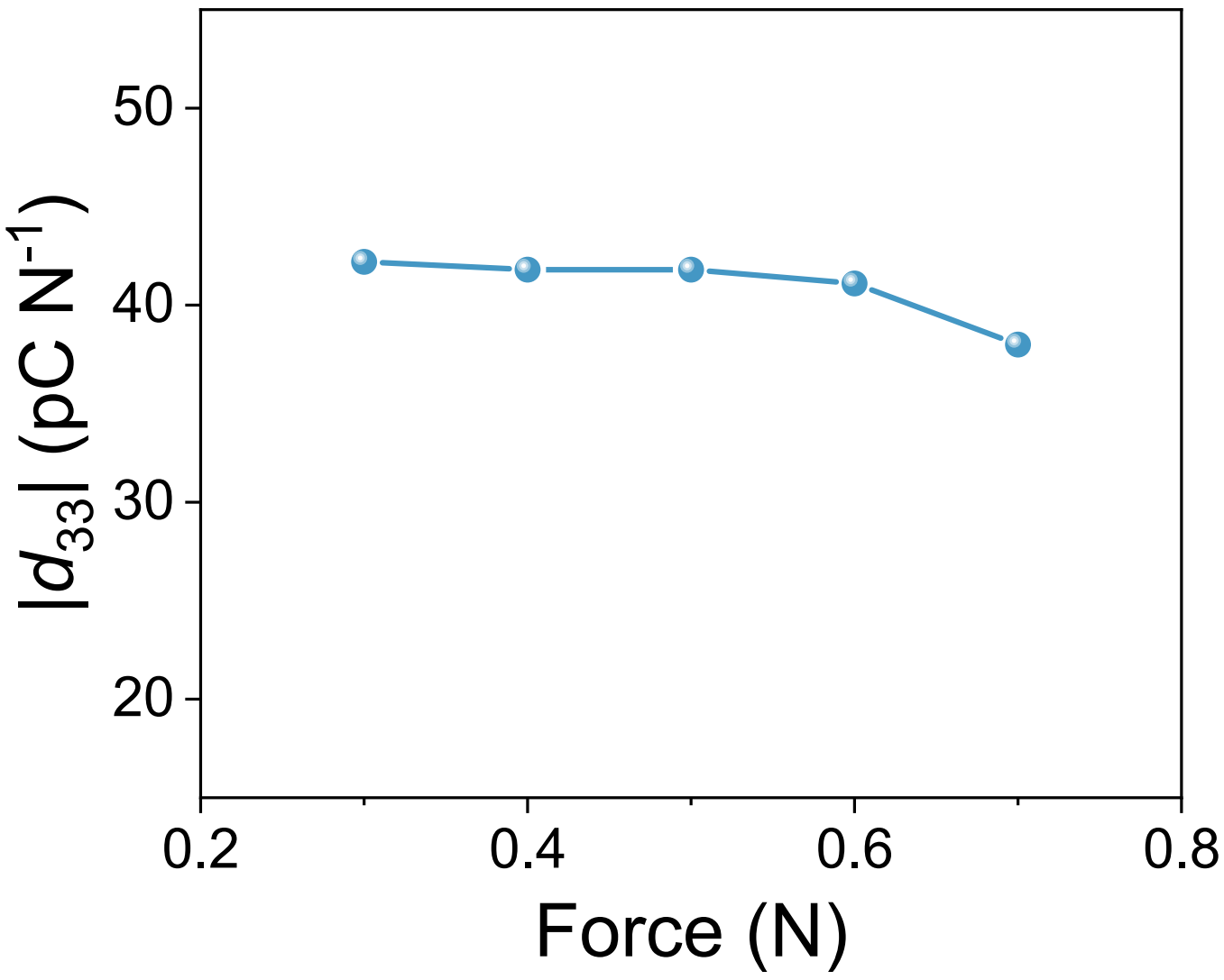

Figure S26. d_{33} measured under different static forces by d_{33} meter (graft P(VDF-TFE) with a grafting density of 1.2%).

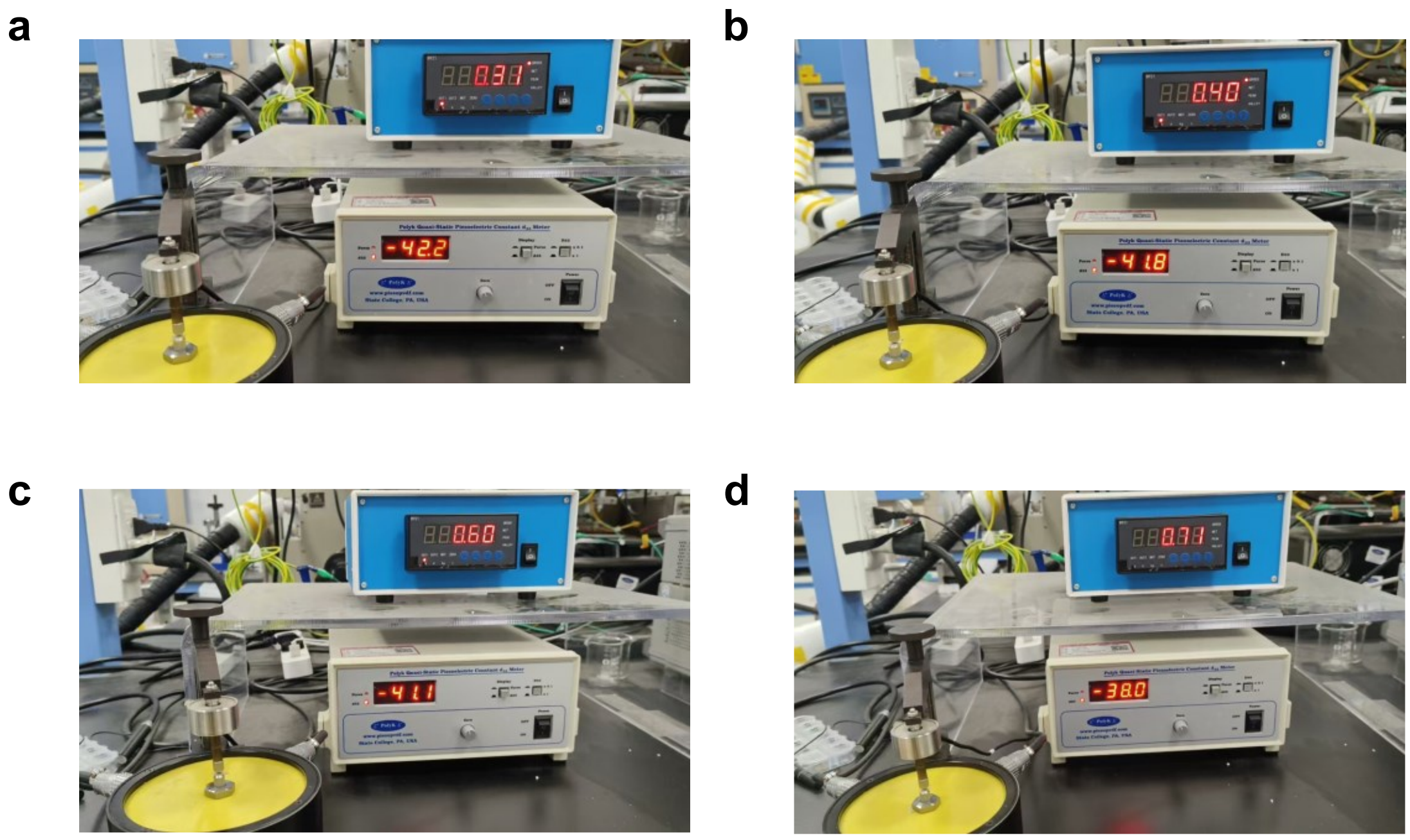

Figure S27. Photo images of d_{33} results measured under different static forces by d_{33} meter. (a) 0.31 N. (b) 0.40 N. (c) 0.60 N. (d) 0.71 N. (graft P(VDF-TFE) with a grafting density of 1.2%).

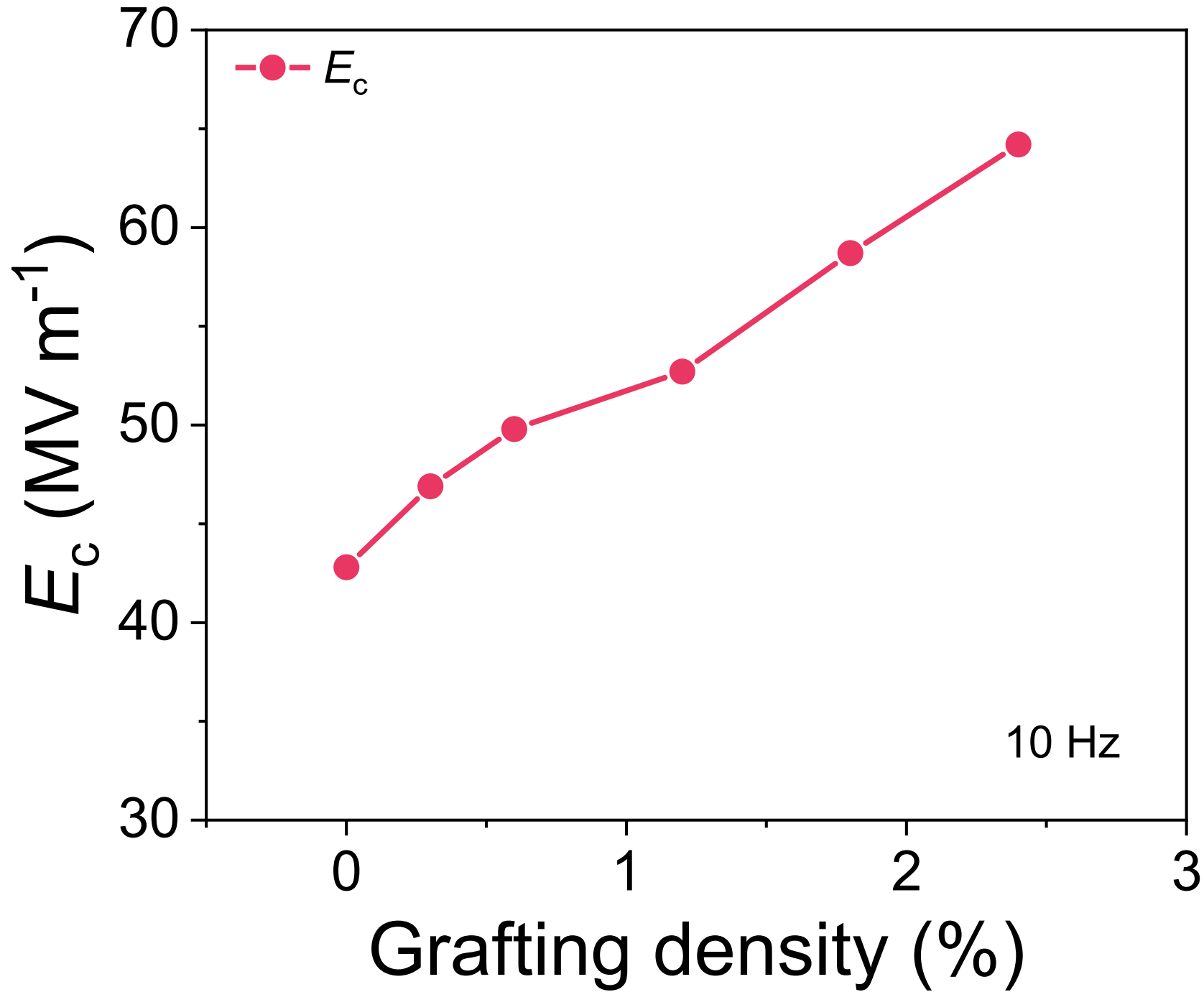

Figure S28. E_c as a function of grafting density in graft P(VDF-TFE) copolymers.

Table S1 Comparison of energy change of the α phase under varied convergence conditions.

Convergence conditions	U (eV)	$\|\delta U/U\|$ (%)
kpoints: 1×1×1	-1371.064	
kpoints:1×4×4	-1373.356	0.17
energy cutoff: 400 eV	-1371.064	
energy cutoff: 500 eV	-1371.236	0.01
iteration convergence of 10^{-5} eV	-1371.064	
iteration convergence of 10^{-6} eV	-1372.847	0.13
with no dispersion corrections	-1371.064	
with dispersion corrections	-1369.817	0.09
Condition 1	-1371.064	
Condition 2	-1371.581	0.03

Condition 1: 1×1×1 kpoints, energy cutoff of 400 eV electronic self-consistent iteration convergence of 10^{-5} eV without dispersion corrections.

Condition 2: 1×4×4 kpoints, energy cutoff of 500 eV electronic self-consistent iteration convergence of 10^{-6} eV with dispersion corrections.

Table S2. Comparison of piezoelectric properties of graft P(VDF-TFE) with other ferroelectric polymers.

Polymers	$|d_{33}|$ (pC N^{-1})	T_c or T_M(°C)	Reference
**Graft P(VDF-TFE) 95/5 mol% (1.2%)**	**41.6**	**133**	**This work**
P(VDF-TrFE) 80/20 mol%	19	136	S1
P(VDF-TrFE) 70/30 mol%	26.2	107	S2
P(VDF-TrFE) 65/35 mol%	31.4	90	S3
P(VDF-TrFE) 52/48 mol%	69	65	S4
P(VDF-TrFE) 50/50 mol%	63.5	65	S5
P(VDF-TFE) 80/20 mol%	12	NA	S6
P(VDF-TFE) 90/10 mol%	9	138.5	S7
P(VDF-TFE) 85/15 mol%	13	130.9	S7
P(VDF-TFE) 81/19 mol%	12	128.5	S7
P(VDF-TFE) 80/20 mol%	12	129.4	S7
P(VDF-TFE) 78/22 mol%	11	134.5	S7
P(VDF-TFE) 74/26 mol%	10	138.7	S7
P(VDF-TFE) 80/20 mol%	16	130.0	S1
Irradiated P(VDF-TFE) 65/35 mol%	69	73	S8
P(VDF-TrFE-CTFE) 64.5/33.1/2.4 mol%	55.4	47	S9

References

[S1] Li, Z., Liao, J., Xi, Z., Zhu, W. & Zhang, Z. C. Influence of steric hindrance on ferro- and piezoelectric performance of poly(vinylidene fluoride)-based ferroelectric polymers. *Macromol. Chem. Phys.* **220**, 1900273 (2019).

[S2] McGinn, C. K., Kam, K. A., Laurila, M.-M., Lozano Montero, K., Mäntysalo, M., Lupo, D. & Kymissis, I. Formulation, printing, and poling method for piezoelectric films based on PVDF-TrFE. *J. Appl. Phys.* **128**, 225304 (2020).

[S3] Katsouras, I., Asadi, K., Li, M., van Driel, T. B., Kjær, K. S., Zhao, D., Lenz, T., Gu, Y., Blom, P. W. M., Damjanovic, D., Nielsen, M. M. & de Leeuw, D. M. The negative piezoelectric effect of the ferroelectric polymer poly(vinylidene fluoride). *Nat. Mater.* **15**, 78-84 (2016).

[S4] Zhu, Z., Rui, G., Li, Q., Allahyarov, E., Li, R., Soulestin, T., Domingues Dos Santos, F., He, H., Taylor, P. L. & Zhu, L. Electrostriction-enhanced giant piezoelectricity via relaxor-like secondary crystals in extended-chain ferroelectric polymers. *Matter* **4**, 3696-3709 (2021).

[S5] Liu, Y., Aziguli, H., Zhang, B., Xu, W., Lu, W., Bernholc, J. & Wang, Q. Ferroelectric polymers exhibiting behaviour reminiscent of a morphotropic phase boundary. *Nature* **562**, 96-100 (2018).

[S6] Wen, J. X. Piezoelectricity and pyroelectricity in a copolymer of vinylidene fluoride and tetrafluoroethylene. *Polym. J.* **17**, 399–407 (1985).

[S7] Fan, X., Sun, M., Li, Z., Chen, Z., Zhou, X., Lu, Q. & Zhang, Z. Tuning piezoelectric properties of P(VDF-TFE) films for sensor application. *React. Funct. Polym.* **180**, 105391 (2022).

[S8] Liu, Y. Q., Tong, H., Li, C., Qi, W., Zhou, H. & Liu, Y. Morphotropic phase boundary in irradiated ferroelectric polymers. *Adv. Mater.* **37**, 2502099 (2025).

[S9] Han, Z., Liu, Y., Chen, X., Xu, W. & Wang, Q. Enhanced piezoelectricity in poly(vinylidene fluoride-co-trifluoroethylene-co-chlorotrifluoroethylene) random terpolymers with mixed ferroelectric phases. *Macromolecules* **55**, 2703-2713 (2022).
